# A Review of the Regulatory Mechanisms of N-Myc on Cell Cycle

**DOI:** 10.3390/molecules28031141

**Published:** 2023-01-23

**Authors:** Hong-Li Li, Lu-Lu Dong, Min-Jie Jin, Qian-Yu Li, Xiao Wang, Mei-Qi Jia, Jian Song, Sai-Yang Zhang, Shuo Yuan

**Affiliations:** 1 Children’s Hospital Affiliated of Zhengzhou University, Henan Children’s Hospital, Zhengzhou Children’s Hospital, Zhengzhou 450018, China; 2 Faculty of Laboratory Medicine, Zhengzhou University, Zhengzhou 450001, China; 3 School of Basic Medical Sciences, Zhengzhou University, Zhengzhou 450001, China; 4 School of Pharmaceutical Sciences, Institute of Drug Discovery & Development, Zhengzhou University, Zhengzhou 450001, China

**Keywords:** *MYCN*, cell cycle, proliferation, neuroblastoma

## Abstract

Neuroblastoma has obvious heterogeneity. It is one of the few undifferentiated malignant tumors that can spontaneously degenerate into completely benign tumors. However, for its high-risk type, even with various intensive treatment options, the prognosis is still unsatisfactory. At the same time, a large number of research data show that the abnormal amplification and high-level expression of the *MYCN* gene are positively correlated with the malignant progression, poor prognosis, and mortality of neuroblastoma. In this context, this article explores the role of the N-Myc, *MYCN* gene expression product on its target genes related to the cell cycle and reveals its regulatory network in promoting tumor proliferation and malignant progression. We hope it can provide ideas and direction for the research and development of drugs targeting N-Myc and its downstream target genes.

## 1. Introduction

Neuroblastoma (NB) is the most common extracranial tumor in children. Nearly half of all NBs occur in infants under 2 years of age [1]. NB accounts for approximately 6–10% of childhood tumors and 5% of tumor mortality in 1-year-old children [1]. According to different high-risk factors, NB can be divided into the low-risk group, intermediate-risk group, and high-risk group [2]. For high-risk patients with NB, the prognosis is still unsatisfactory even if various intensive treatment options are combined [3,4]. It is worth noting that a large number of clinical data have shown that patients with high-risk neuroblastoma are often accompanied by abnormal amplification and high-level expression of the *MYCN* gene [5,6], suggesting that the *MYCN* gene plays an important role in tumor malignancy and development. Therefore, we took neuroblastoma as an entry point to summarize the potential mechanism of N-Myc regulation on the cell cycle, and also considered the regulation network in other tumor cells, in an attempt to complete the regulatory network of N-Myc on the cell cycle.

The *MYCN* gene is an important member of the MYC family. The MYC family is a group of oncogenes that was discovered earlier and has been studied extensively. It consists of three members: *MYC*, *MYCN*, and *MYCL*. All of them belong to the basic helix-loop-helix leucine zipper (bHLHLZ) DNA binding protein superfamily, and their products are nuclear DNA binding proteins [7,8], which usually act as transcriptional regulators and affect various processes such as the cell cycle, differentiation, angiogenesis, etc., by regulating a variety of genes [9]. Among them, the *MYCN* gene has certain temporal and spatial expression specificities. It is usually expressed in pre-B cells, kidneys, forebrain, hindbrain, and intestines during embryogenesis, and is highest in the developing brain [10].

**Figure 1:**
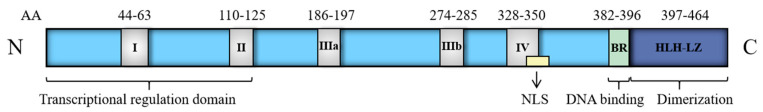
Structure of N-Myc. Silver boxes represent Myc homology Box (MB) I–IV. Green Box represents Basic Region (BR), which is DNA-binding domain. Dark blue Box represents Helix-Loop-Helix-Leucine Zipper (HLH-LZ), associated with MYC dimerization. Yellow box represents nuclear localization signal (NLS). Blue bottom frame is region of N-Myc with relatively low disorder score.

N-Myc, the expression product of the *MYCN* gene, is a transcription factor with 464 amino acids, composed of MYC Boxes (including MBI, MBII, MBIIIa, MBIIIb, MBIV), the nuclear localization signal (NLS), the highly conservative basic region (BR), and the helix-loop-helix-leucine zipper (HLH-Zip) [11]. Among them, MBI and MBII are located in the N-terminal transcriptional regulatory region (TAD). MBIIIa, MBIIIb, MBIV, and NLS are sequentially distributed in the central region. MBIV and NLS have a partially overlapping region. The polypeptide at the C-terminus is the highly conserved BR region and HLH-Zip. These regions are closely related to the interaction of N-Myc with other proteins and the regulation of gene transcription [11] (Figure 1).

N-Myc can regulate the expression of various target genes that are related to the cell cycle to affect the proliferation and development of tumor cells. The methods of regulation can be summarized as the following: First, the most classic regulation method is that N-Myc binds to the HLH-Zip of the chaperone MYC-associated protein X (MAX) in the nucleus to form a complex through the HLH-Zip structure, and acts on the E-box region that exists on the regulatory sequence of its target gene to regulate the expression of target genes [12]. N-Myc preferentially combines with E-box CATGTG and classic CACGTG. However, under the conditions of *MYCN* amplification, its specificity is reduced, and it can bind to other non-classical E-box motifs, including CTTTG, CACTTG, and CAACTG) [13]. It should be noted that although the E-box region on the gene is considered to be the classic scope of the MYC family, the E-box region where c-Myc can act does not mean that N-Myc can also act. In addition to MAX, N-Myc can also interact with WD repeat domain 5 (WDR5), which is a subunit of the histone H3K4 methyltransferase complex [14], H3K9me3/me2 demethylase, lysine demethylase 4B (KDM4B) [15], lysine demethylase 1A (KDM1A/LSD1) [16], etc., to regulate gene expression. Second, N-Myc can act independently on the E-box region of the transcription promoter of the target genes and directly regulate their expression [17]. Third, under the condition of gene co-expression, when both p53 and *MYCN* are expressed at high levels, N-Myc (MYC BOX II/IIIa region) can interact with the C-terminus of the tetramerized p53 protein (only tetrameric p53 can bind to chromatin) to form a complex, and this effect has nothing to do with the formation of N-Myc and MAX heterodimer mentioned above. The complex can regulate gene expression by acting on the E-box region or p53 response elements (p53-Res) [18]. Fourth, the antisense mRNA transcribed by *MYCN* itself can regulate its own expression by acting on the gene or its transcribed mRNA [19], indirectly affecting the expression of downstream genes. Therefore, N-Myc can affect the expression level of cell cycle-related target genes in a variety of ways, which is closely related to cell malignant proliferation.

Cyclins or the cyclin-dependent kinase (CDK) play a vital role in cell cycle regulation [20]. Cyclins are synthesized and degraded periodically as the cell cycle progresses, and they all have a conservative sequence (cyclin box) composed of approximately 100 amino acids, which mediates the combination of cyclin and CDK to form a complex and regulate the cell cycle [21]. CDK must be combined with cyclin to exhibit kinase activity. Therefore, the orderly appearance and degradation of cyclin can effectively regulate the activity of CDK, thereby initiating or regulating events such as DNA replication, mitosis, and cytokinesis [22]. The cyclin-dependent kinase inhibitor (CKI) can interact with cyclin and the CDK complex and inactivate it by changing the structure of the active site of CDK. CKI includes two families, the CDK-interacting protein/kinase inhibition protein (CIP/KIP) and inhibitors of CDK (INK), and is primarily involved in the regulation of G1 and S phases [23]. In addition, during the progression of the cell cycle, cyclin and CDK need to be accumulated, combined, activated to complete their normal physiological functions, and finally, degraded. The Skp1-Cullin 1-F Box (SCF) and the anaphase promotion complex (APC), both of which are ubiquitin ligase, link ubiquitin to G1/S and M phase cyclins and CKIs to degrade, thereby regulating the cell cycle [23].

In the beginning, we comprehensively referred to several papers on transcriptomic analysis of *MYCN* regulatory gene expression and screened a wide range of potential target genes [24]. However, transcriptomic analysis lacked strong evidence to prove the direct targeting of *MYCN* for these genes, so we further referred to articles related to experimental research to support the results.

## 2. The Target Genes of N-Myc

**Figure 2:**
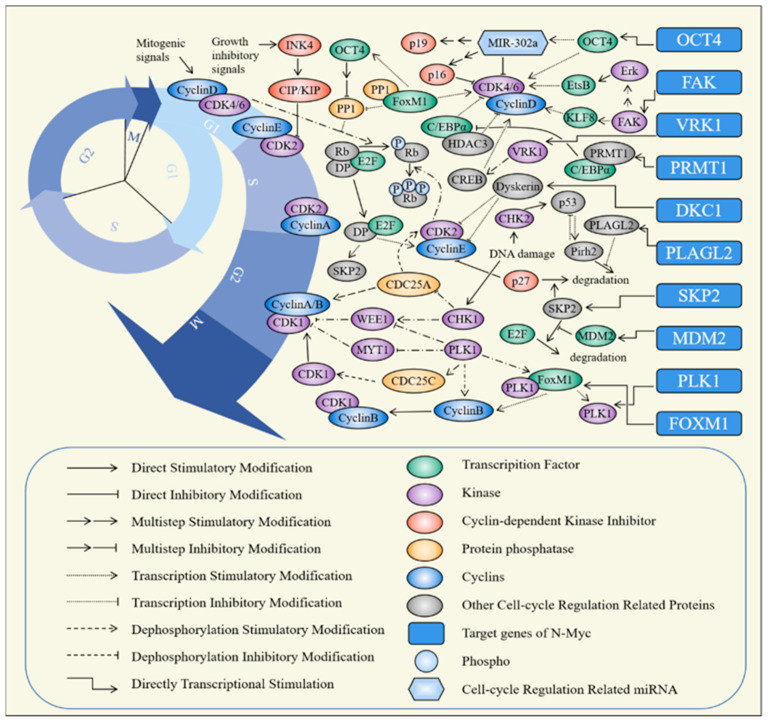
The mechanism of N-Myc target genes regulating cell cycle progression. The ten target genes listed on the right side can be divided into three parts. First, the expression products of genes such as *POU5F1*, *PTK2*, *PRMT1*, *MDM2*, *FOXM1*, and *DKC1* can act as or bind to transcription factors to regulate the expression of cell cycle-related genes (primarily including CDKs and Cyclins). Second, they serve as kinase to regulate the activity of proteins participating in cell cycle, such as PTK2, VRK1, and PLK1. Third, they play a vital role in balancing the stability of cell-cycle-related proteins, such as SKP2, MDM2, and PLAGL2.

The target genes of N-Myc below are closely related to the cell cycle, and some of their products can act as transcription factors or combine with them to directly affect the expression of the key factors of the cell cycle, such as Cyclins and CDK, while some of them can regulate the activity and stability of cell-cycle-related proteins by exerting the activity of enzymes. In general, we screened 10 N-Myc target genes and listed them in the order of cell cycle progression (G1-M) according to their main mode of action. At the same time, the regulatory mechanisms of these target genes on the cell cycle are summarized (Supplementary Materials Table S1) and form a downstream signal regulatory network of N-Myc (Figure 2).

### 2.1. POU5F1

POU class 5 homeobox 1 (POU5F1, also knowns as OCT4) is a homeodomain transcription factor of the Pit-Oct-Unc (POU) family, which is necessary for inducing the pluripotency of human and mouse somatic cells [25]. Pou5f1 can bind to the octamer motif binding site in the enhancer region or target promoter to activate the transcription and expression of target genes [26]. Bioinformatics analysis shows that *POU5F1* is overexpressed in a variety of cancers [27].

Experiments have shown that endogenous N-Myc binds directly to the E-box site in the *POU5F1* promoter to enhance its transcription. Furthermore, Pou5f1 binds to the first intron of *MYCN* to promote *MYCN* transcription, so the two can form a positive feedback loop [28].

The overexpression of *POU5F1* in BE(2)-C human NB type I cells can promote cell proliferation and increase its colony formation rate [29]. There are many mechanisms for Pou5f1 to regulate the cell cycle.

First, in adult stem cells or cancer cells, Pou5f1 up-regulates the expression of *CCND* by binding to the octamer motif in the *CCND* promoter region, thereby promoting the G1/S transition [30,31,32].

In addition, Pou5f1 can bind to the conservative promoter of miR-302 and up-regulate its expression [33]. miR-302 (over a certain threshold concentration) simultaneously suppressed the activities of CDK2 and cyclin D1/D2 to inactivate both complexes (cyclin D-CDK4/6 and cyclin E-CDK2), thereby blocking both G1-S transition pathways. miR-302 suppresses BMI-1 to slightly stimulate p16Ink4a/p14ARF expression, which binds to CDK4/6 and reduces its activity, thereby inhibiting the phosphorylation of retinoblastoma protein RB and eventually blocking a series of genes to prevent the entry of the S phase [34]. 

According to the classical model, in the early and middle G1 phase, Pou5f1 down-regulates the protein phosphatase-1 (PP1) by promoting the expression of PP1 inhibitor nuclear inhibitor of protein phosphatase 1 (*NIPP1*) and *CCNF*, which inhibit the dephosphorylation of RB by PP1 [35]. Pou5f1 can also up-regulate CDK4/6-Cyclin D to promote the hypo-phosphorylation of RB. In mammalian cells, the physiological early 2 factor (E2F) exists as an E2F/dimerization partner (DP) heterodimer. E2F binds to DNA with DP proteins through an E2 recognition site and regulates gene transcription. RB can bind to E2F/DP to form a ternary complex to inhibit E2F activity [36]. When RB is hypo-phosphorylated, part of RB is released, restoring the transcriptional activity of E2F to up-regulate *CCNE1/2* expression [37]. Cyclin E binds to and activates CDK2, which drives the hyperphosphorylation of RB and causes stronger effects, thereby driving the restricted point passage and increasing the transcription required for S phase entry. [37]. In addition, A new study suggests that CDK4/6-Cyclin D can only monophosphorylate the RB protein, leaving it still connected to E2F and inhibiting its activity. An unknown event can activate Cyclin E-CDK2 and further phosphorylate RB, thereby activating E2F. The mechanism of this new model remains to be further studied [37,38]. 

In addition, RB can directly bind to the central domain of the forkhead box M1 (*FOXM1*), thereby indirectly inhibiting the transactivation domain of *FOXM1* and inhibiting the effect of FoxM1 as a transcription factor up-regulating the expression of *POU5F1* [39].

In the G2/M phase, Pou5f1 can inhibit CDK1 activation by inhibiting the cell division cycle 25 (Cdc25) [38], resulting in a prolonged duration of the G2 phase, which is conducive to subsequent genome integrity checks and reducing chromosomal segregation [40].

Research shows that silencing *POU5F1* significantly reduced the expression of the deacetylase the silent information regulator factor 2-related enzyme 1 (*SIRT1*), resulting in increased acetylation of p53 at K120 and K164, thereby maintaining the stability of p53 [38]. In addition, Pou5f1 can bind to the promoter region of integrin subunit α6 (CD49f), whose overexpression is sufficient to regulate cellular proliferation via regulation of the PI3K/AKT/p53 pathway [41]. *p21* is a downstream target of p53, which can inhibit the activation of CDKs, leading to G1 and G2 blockade. Pou5f1 can also inhibit the activity of *p21* by directly binding to the *p21* promoter region or indirectly up-regulating DNA methyltransferase 1 (*DNMT1*), which is a major DNA methyltransferase responsible for maintaining methylation status during DNA replication [42].

### 2.2. PRMT1

Protein arginine N-methyltransferase 1 (PRMT1) is the most important asymmetric arginine methyltransferase in the human body. It plays a role in transcriptional co-activation by dimethylating histone H4 at R3 (H4R3me2as) [43]. PRMT1 is highly expressed in a variety of tumors and is positively correlated with tumor growth, progression, and poor prognosis [44,45,46].

N-Myc can up-regulate the expression of *PRMT1* by acting on the region near the transcription promoter of the *PRMT1* gene [47]. At the same time, the down-regulation of *MYCN* mRNA caused by the knockdown of *PRMT1* suggests that PRMT1 may regulate the expression of *MYCN* at the transcriptional level [48]. In addition, studies have shown that PRMT1 enhanced the stability and expression of *MYCN* by methylating N-Myc protein at R65, which is related to CDK-mediated phosphorylation of N-Myc at S62 [48].

In breast cancer cells, PRMT1 can interact with CCAAT/enhancer-binding protein alpha (C/EBPα), a member of the leucine zipper transcription factor family, and methylate it at both R35 and R156 (the most critical sites) residues. The methylation of C/EBPα prevents its interaction with the inhibitor histone deacetylase 3 (HDAC3) and reduces the formation of the C/EBPα-HDAC3 complex, which has an inhibitory effect on Cyclin D1, thereby promoting the proliferation of tumor cells [45]. C/EBPα can act on the promoter region of the *CCND1* gene to negatively regulate its expression. At the same time, C/EBPα can also exert a stronger inhibitory effect by recruiting HDAC3 to the promoter region of *CCND1* [45].

In addition, studies have shown that PRMT1 can regulate the expression of a series of cell-cycle-related genes, including *CCNA2*, *CCNB1*, *CCND1*, *CCNE2*, *CDK6*, *CDC20*, and *CDC23* [45]. The mechanism is still unclear, but it proved that PRMT1 was closely related to cell cycle regulation. However, in NB, PRMT1 promotes cell survival by up-regulating the expression of activating transcription factor 5 (*ATF5*) and inhibiting apoptosis, instead of regulating the cell cycle [49].

### 2.3. VRK1

Vaccinia-related kinase 1 (VRK1) is a member of the Ser/Thr kinase family. VRK1 can phosphorylate a variety of transcription factors including p53, and it can also cooperate with the c-Jun NH2-terminal kinase (JNK) pathway through the phosphorylation of c-Jun and activating transcription factor 2 (ATF2) to participate in cellular stress response [50]. VRK1 is highly expressed in a variety of tumor cells and is positively correlated with tumor cell proliferation, tumor progression, and poor prognosis [51,52,53,54].

*VRK1* has been identified as the transcription target of *MYCN*, its gene transcription promoter region contains an E-box region, and its expression can be up-regulated by *MYCN*. At the same time, the analysis showed that in *MYCN*-amplified NB cells, the binding site of N-Myc presented a hypomethylated state (the degree of methylation is negatively correlated with the degree of gene expression), proving that *VRK1* is regulated by *MYCN*. In addition, the down-regulation of *VRK1* can also down-regulate the expression of *MYCN*, suggesting that there may be a positive feedback mechanism between the two [53].

High expression of *VRK1* promotes the proliferation of NB cells [53]. Experiments showed that the proliferation of NB cells depends on the expression of VRK1. Observed via mRNA and immunohistochemistry, in NB and patient-derived tumor xenograft (PDX)-derived cells, there is a strong positive correlation between the expression of VRK1 and that of the proliferation marker Ki67, as well as the mitotic index in the tumor. Concomitantly with this, a moderate knockdown of *VRK1* will induce the down-regulation of cell-cycle-progressing protein levels (such as Cyclin D1 or MDM2) and lead to an increase in cell cycle inhibitors (such as p53 and its target p21) [53]. From the late G1 phase to the early S phase, VRK1 phosphorylates the cAMP-response-element binding protein (CREB), which causes CREB to bind to the cAMP response element (CRE) of the *CCND1* promoter and activate its transcription [55]. In liver cancer cells, the knockdown of *VRK1* also up-regulates the expression of p27, which, in turn, causes cell G1/S phase arrest [56]. In addition, VRK1 is also necessary for exiting G0 and entering G1 [57].

In esophageal squamous cell carcinoma, the knockdown of *VRK1* leads to the down-regulation of the barrier-to-autointegration factor 1 (BANF1) [58]. BANF1 plays an important role in mitotic nuclear reorganization and directly affects cell proliferation. In addition, BANF1 can be used as a high-affinity substrate of VRK1 to be catalyzed and phosphorylated by VRK1, thereby weakening the interaction between BANF1 and DNA, destroying the connection between DNA and the nuclear membrane and maintaining the normal process of the cell cycle, resulting in a change in the process of cell mitosis [59,60]. Studies have shown that histone H2A T120 is phosphorylated by hVRK1 (human VRK1) in and around the *CCND1* promoter. H2A T120 phosphorylation antagonizes H2A K119 ubiquitination and promotes H3 K4 methylation, thereby up-regulating *CCND1* and promoting the oncogenic transformation of cells [61].

In head and neck squamous cell carcinoma, the VRK1 protein is positively correlated with several proliferation-related proteins including CDK2, CDK6, Cdc2, Cyclin B1/A, topoisomerase II (TOP2), survivin, and Ki67, suggesting that VRK1 can regulate the cell cycle [54]. VRK1 can act on the promoter regions of *CDK2* and *BIRC5* (also known as *survivin*) genes, up-regulate their expression, and regulate the cell cycle [54].

### 2.4. SKP2

The S-phase kinase-associated protein 2 (*SKP2*) gene is located in the 5pl3 region of the human chromosome, and the protein encoded by it is composed of 436 amino acid residues, also known as the p45 protein. *SKP2* is sequentially linked by the F-box sequence, linker sequence, and protein–protein interaction module [62,63]. *SKP2* is overexpressed in a variety of tumors and is positively correlated with tumor progression and poor prognosis [64,65,66].

Studies have shown that in *MYCN*-amplified NB cells, N-Myc can increase the activity of the *SKP2* gene promoter and promote its expression by acting on the classic or non-classical E-box region of the *SKP2* gene transcription promoter region [67]. In *MYCN*-amplified NB cells, N-Myc eliminates the repressive pRB-E2F transcription factor 1 (E2F1) complex bound to the *SKP2* promoter by inducing CDK4 and up-regulates the expression of *SKP2* [68].

The *SKP2* gene contains a functional E2F response element (hSRE2), which is involved in the activation of the *SKP2* promoter function. It is also required for the high-level expression of the *SKP2* gene in many human tumor cell lines [69]. At the same time, the expression level of *SKP2* is also regulated by RB. The combination of RB and E2F can inhibit the transcription of *SKP2*. In the G0 phase and the early stage of the G1 phase, RB can inhibit the expression of the Skp2 protein by keeping Skp2 and APC/CCdh1 in close proximity [70]. Both of these effects will keep Skp2 at a low level. In the late stage of the G1 phase, RB is phosphorylated and releases E2F and APC/CCdh1, causing *SKP2* to be induced to be transcribed, and at the same time, its degradation efficiency is reduced, causing the cell to pass through the R point and enter the S phase [71].

A large number of studies have shown that there is an automatic induction circuit of Skp2. In this loop, the ectopic expression of *SKP2* triggers the degradation of p27, leading to the activation of Cyclin E-CDK2, which was originally in an inhibited state by binding to p27. The activated CDK2 causes the phosphorylation of RB, and the release of E2F leads to the increased expression of *SKP2* [71,72,73]. Mitogenic stimulation initiates the Skp2 automatic induction circuit by inducing Cyclin D1, activating CDK4/6, and inactivating RB [74]. Exogenous anti-mitogens, such as hyaluronic acid, may close the circuit by inhibiting the effects of Cyclin D1 and CDK4/6 [75].

A new mechanism for *SKP2* to drive cell-cycle progression has been proposed. In the late stage of the G1 phase, activated Cyclin E-CDK2 phosphorylates Erα, triggering ERαSCF^Skp2^ binding and E2F1 transactivation. E2F1 further induces Cyclin E, Cyclin A, and Skp2, and drives the entry of the late G1 and S phases [76].

Skp2 is primarily related to the G1/S process. It targets cell cycle inhibitors (such as p27^Kip1^, p21^Cip1^, etc.) for ubiquitination and degradation, thereby promoting the cell cycle process [62,63,77]. It is a part of the SCF ubiquitin ligase E3 complex (SCF) and can link ubiquitin and induce the degradation of S phase kinase cyclin A and CDKI. Knockdown of *SKP2* can significantly inhibit the growth and proliferation of *MYCN*-amplified or non-amplified NB cells. Studies have shown that the G1/S phase arrest of NB cells caused by the down-regulation of the Skp2 protein is positively correlated with the stability of the p27 protein. If the accumulation of p27 caused by the down-regulation of *SKP2* is inhibited, the degree of cell cycle arrest will be reduced, indicating that the down-regulation of *SKP2* causes the accumulation of p27, which causes tumor cells to arrest in the G1/S phase [63,67].

### 2.5. PTK2

Protein tyrosine kinase 2 (PTK2, also called adhesion focus kinase, FAK) is a non-receptor cytoplasmic protein tyrosine kinase, which participates in the control of many signaling pathways by integrating signals from integrin and growth factor receptors, affecting cell proliferation, viability, movement, and survival. It has become the target of a variety of malignant tumors [78].

There are two N-Myc binding sites in the *PTK2* promoter sequence. The electrophoretic mobility shift assay and chromatin immunoprecipitation (ChIP) proved that N-Myc bound to the E-box in the *PTK2* promoter [79]. Dual luciferase analysis showed that the activity of the *PTK2* promoter was significantly increased in *MYCN*-amplified NB cells. A polymerase chain reaction (PCR) and Western Blot confirmed that mRNA and protein levels were elevated in NB cell lines along with elevated *MYCN* levels. In summary, *MYCN* can up-regulate *PTK2* gene expression [79].

In *MYCN*-amplified NB cells, after inhibiting the expression of *PTK2* with two PTK2 small-molecule inhibitors, the proliferation of tumor cells was significantly reduced, the percentage of cells in the G1 phase was significantly increased, and that in the S phase was decreased [80]. It showed that inhibiting PTK2 can inhibit the proliferation of NB cells and cause the cells to fail to pass the cell cycle and arrest in the G1 phase [80].

PTK2 is an important cell-signaling scaffold. When PTK2 is phosphorylated, it can activate downstream pathways, such as sarcoma family kinases (SFKs) and extracellular signal-regulated kinase (ERK), and then participate in the regulation of cell cycle processes [81]. In fibroblasts, PTK2 can enhance the activity of the transcription factor E26 transformation-specific B (EtsB) and the *CCND1* promoter, up-regulating the expression of *CCND1* [82]. Ets family transcription factors are specific downstream substrates of ERK. Experimental data showed that PTK2 regulated the transcription of *CCND1* primarily through the activation of the ERK pathway [82]. As a target gene of PTK2, Krüppel-like transcription factor 8 (*KLF8*), a member of the family of transcription factors, was positively regulated by PTK2 [83]. The promoter sequence of *CCND1* has been identified as the target of KLF8. *CCND1* can be directly activated by the combination of KLF8 and GT box A, or by inhibiting the potential inhibitory regulator of Cyclin D1 [83]. In glioblastoma cells, high *PTK2* expression can increase the expression of *CCND1* and *CCNE*, reduce the expression of *CDKN1B* (*p27^Kip1^*) and *p21^Waf1^*, and enhance the activity of CDK4, thereby promoting the G1/S phase transformation of glioblastoma cells [84].

### 2.6. DKC1

Dyskerin pseudouridine synthase 1 (*DKC1*) is a conservative X-linked gene encoding the RNA-binding protein Dyskerin (pseudouridine synthase). Dyskerin is an important part of telomerase. Dyskerin can bind to and stabilize small nucleolar RNAs-H/ACA box snoRNAs, thereby directing rRNA modification and playing a role in the processing of rRNA precursors [85]. In clear cell renal cell carcinoma (ccRCC) [86], glioma [87], NB [88], and liver cancer [89], *DKC1* expression is up-regulated and promotes tumor progression, but it can also act as a tumor suppressor [90].

Experiments have shown that in the BE(2)-C cell line, N-Myc interacted with its chaperone MAX to form a complex, acting on a site near the *DKC1* transcription promoter, which includes a non-canonical E-box region containing a CpG island downstream of the promoter, and two canonical E-box regions further downstream but still within the first intron region. It can up-regulate the expression of the *DKC1* gene [88].

The ribosomal stress caused by the down-regulation of Dyskerin in NB with siRNA is the main reason for the stagnation of tumor cell proliferation [88]. In glioma cells, Dyskerin negatively regulates the expression of *CDK2* and *CCNE2*, leading to G1 arrest [87]. In mouse thymocytes, the genetic interaction between *DKC1* and p27 is required for limited cell cycle progression [91].

However, in pituitary tumors, impaired DKC1 function can affect the translation of specific mRNAs containing internal ribosome entry site (IRES) elements, including tumor suppressor p27. The p27 IRES element mediates the assembly of the 48S translation pre-initiation complex, thereby promoting the occurrence of pituitary tumors [90].

### 2.7. MDM2

The mouse double minute-2 (*MDM2*) gene belongs to the RING-finger protein family and is widely known as an oncogene. It is amplified in a variety of human cancers including NB [92,93] and is involved in a variety of key cell growth regulation processes [94].

In NB, N-Myc directly binds to the E-box in the *MDM2* promoter and promotes the transcription of *MDM2* [95]. In addition, when MDM2 transfers from the nucleus to the cytoplasm, it combines with the AU-rich elements in the *MYCN* 3′ untranslated region (3′-UTR) to regulate the stability of *MYCN* mRNA and its translation, so the interaction between *MYCN* and *MDM2* forms a positive feedback regulatory loop [96].

In normal cells, the overexpression of *MDM2* can induce G1 phase arrest, but in many cancer cells, including cells overexpressing *MDM2*, the arrest in the G1 phase is not apparent. Mdm2 contains three growth-inhibitory domains, the overexpression of which can inhibit the proliferation of normal cells, but cancer cells overexpressing *MDM2* can make the growth-inhibitory domains unable to perform their normal functions through a variety of ways, thereby evading the G1 phase block [97,98]. Experiments have shown that the Mdm2 protein mediated the ubiquitination and degradation of p53 and inhibited the function of p53 to activate the transcription of target genes, thereby reversing p53-mediated cell cycle arrest [99,100,101]. The growth suppressor p14/p19 interacts with Mdm2 to inhibit Mdm2-mediated ubiquitination and degradation of p53, thereby restoring the regulation of the cell cycle by p53 [102,103,104]. Mdm2 can also act as a bridge between RB and p53, forming an RB-MDM2-p53 trimer, thereby preventing the degradation of p53 [105].

In prostate cancer cells, Mdm2 competes with E3 ubiquitin ligase SCF^Skp2^ to bind to E2F1 and inhibits the ubiquitination of E2F1, thereby up-regulating the protein level of E2F1 [106]. E2F1 is considered a carcinogen due to its activity promoting cell cycle progression. In the early stage of G0/G1, RB binds to E2F1 to inhibit its transcriptional function [106,107]. When RB is phosphorylated, it can dissociate from E2F1, thereby activating the transcription of downstream target genes related to the cell cycle and enabling cells in the late G1 phase to initiate cell cycle progression [106,107].

### 2.8. FOXM1

FoxM1 belongs to the mammalian fox family transcription factor and has homology in its winged helix DNA binding domain [108]. FoxM1 plays a vital role in ensuring the fidelity of the cell division process. Inhibition of FoxM1 activity can lead to serious abnormalities during mitosis, such as frequent chromosome segregation, cytokinesis defects, and overt aneuploidy [109]. *FOXM1* is up-regulated in a variety of cancers and plays a carcinogenic role in tumorigenesis [110].

Studies have shown that N-Myc can directly bind to the promoter of *FOXM1* and up-regulate the expression of *FOXM1* at the transcriptional level in NB cells [111].

FoxM1 is a pro-proliferation transcription factor that promotes cell cycle progression during the transitional phase of G1/S and G2/M [112]. In NB, knocking down *FOXM1* can cause a significant increase in the proportion of cells in the G1 phase and a decrease in the S phase [113]. Many studies have shown that FoxM1 directly or indirectly regulated the activation of target genes, such as *CCND*, *CDK4,* and *CCNE*, that *CDK2* induces cells to enter the S phase, Cyclin A-CDK1 is involved in the G2/M transition, and Cyclin B-CDK1 induces the entry of the M phase and promotes the process of mitosis [109,114,115,116]. FoxM1 up-regulates *CCNB1* and *CCND1* expression by directly activating their corresponding promoters [117]. In addition, some key mitotic regulators, such as Cdc25B, polo-like kinase 1 (PLK1), aurora kinase B (AURKB), and centromere protein F (CENPF), are also regulated by FoxM1 [118]. Together, these indicate that FOXM1 regulates cell cycle progression primarily by regulating the expression of cell-cycle-related proteins. 

FoxM1 and PLK have mutual regulatory effects. In the S/G2 phase, FoxM1 is phosphorylated by CDK1, which is the key to the interaction between PLK1 and FoxM1. In the G2/M phase, PLK1 combines with FoxM1 and directly phosphorylates it, thereby activating it to promote the expression of downstream mitotic regulators, including PLK1 itself [119]. This regulation forms a positive feedback loop, ensuring an orderly mitosis process [119].

### 2.9. PLK1

PLK1 is an important member of the serine/threonine protein kinase family, and its expression is elevated in a variety of human cancers [120]. It can antagonize apoptosis and increase the invasiveness of cancer cells and is positively correlated with poor prognosis [120]. In the DNA damage response, overexpressed *PLK1* triggers the activation of CDK1 in a Cdc25A-dependent manner, and the activated CDK1 covers the checkpoint with damaged DNA during the phase transition, causing tumorigenesis [121].

In NB cells with *MYCN* amplified, N-Myc can directly activate *PLK1* to transcribe and promote its expression [122]. PLK1 specifically binds to SCF^Fbxw7^ ubiquitin ligase to phosphorylate it and promotes its ubiquitination and proteasome degradation, thereby resisting the Fbxw7-mediated degradation of N-Myc, enhancing the stability of N-Myc protein, and constituting a positive feedback loop [122].

The expression of *PLK1* is cell cycle dependent [123]. PLK1 can promote cell cycle progression by regulating multiple steps in the process of mitosis [124]. PLK1-dependent phosphorylation plays a critical role in mitotic spindle formation at the onset of mitosis [125]. PLK1 also controls mesoscopic maturation and is a necessary kinase for kinesin spindle protein (Eg5)-dependent separation of centrosome [126,127].

During the G2/M transition, Cdc25C is phosphorylated and activated by PLK1 and then activates CDK1 by dephosphorylating it, promoting the formation of the Cyclin B1-CDK1 complex and ensuring mitotic entry [128]. Secondly, PLK1 catalyzes the phosphorylation of Wee1 at S53, leading to the E3 ubiquitin ligase-dependent degradation of Wee1. Furthermore, PLK1 catalyzes the phosphorylation of myelin transcription factor 1 (Myt1) at S426, leading to the inhibition of its kinase activity. Both chemical modifications could promote the activation of CDK1 [128,129,130]. PLK1 further phosphorylates Cyclin B1 at S133 to promote the translocation of the Cyclin B1-CDK1 complex to the nucleus, thereby triggering the G2/M transition [131].

PLK1 plays a vital part in regulating sister chromatid separation. During the prophase, PLK1 phosphorylates the SA2 subunit of cohesins, resulting in the dissociation of a large number of cohesins from sister chromatids [132]. A small fraction of the cohesins remains on the sister chromatids, which is conducted by the interaction of protein phosphatase 2A (PP2A) with shugoshin 1 (Sgo1), to ensure chromosomal pairing until the end of metaphase [132,133,134]. Early mitotic inhibitor-1 (Emi1) binds to the Cdc20 subunit of APC/Cdc20 and prevents it from binding to its substrate separation enzyme [135]. PLK1 causes the phosphorylation of Emi1 and its degradation, thereby promoting metaphase and activating APC/C [135,136]. In addition, PLK1 directly phosphorylates and activates APC/C [137]. Activated APC/C causes the activation of the isolated enzyme, which in turn causes the sister chromatids to move toward the poles, leading to anaphase [137,138]. In addition, PLK1 is essential to error-free chromosome segregation by regulating the interaction between cytoplasmic linker protein 170 (CLIP-170) and microtubules [139].

PLK1 has an important effect on initiating cytokinesis. PLK1 directly binds and phosphorylates the centralspindlin subunit HsCYK4 at S157 during the formation of the intermediate zone, promoting the localization of the Rho-GTPase exchange factor (ECT2) in the intermediate zone to form a cleavage trench, marking the beginning of cytokinesis [140]. The initiation of cytokinesis is related to the formation of the polycomb repressive complex 1 (PRC1) complex [141]. CDK1 phosphorylates PRC1 and prevents PLK1 from binding to PRC1 [141]. In addition, PLK1 negatively regulates PRC1 by directly phosphorylating PRC1 at T602 to prevent premature cytokinesis [142].

### 2.10. PLAGL2

Polymorphic adenoma-like protein 2 (PLAGL2) is a zinc finger protein of the PLAG gene family. There are seven C2H2 zinc finger domains at the N-terminus, which are highly conserved and can bind to DNA and enable the transcription factor PLAGL2 to activate the transcription of specific genes [143]. PLAGL2 is related to a variety of malignant tumors, including lipoblastoma, hepatocellular carcinoma, glioma, colorectal cancer, and acute myeloid leukemia [144,145,146,147].

PLAGL2 promotes the transcription of *MYCN* by directly binding to specific sequences upstream of the coding sequence (CDS) of the *MYCN* gene, and N-Myc regulates the transcription of *PLAGL2* through binding to five N-Myc E-boxes in the *PLAGL2* promoter region, which forms a positive feedback regulation [148].

Experiments showed that the mRNA level of p53 family member *TP73* was significantly increased in the PCR array screening of *PLAGL2* expression, suggesting that PLAGL2 is involved in inducing the transcriptional activation of *TP73* [149]. In cells expressing *PLAGL2*, it was observed that the expression levels of *TP73* and its downstream target cell cycle inhibitors p21 and p57 increased [149]. In U937 cells, the expression of *PLAGL2* inhibited cell proliferation and induced the G1 phase arrest and a small degree of arrest in the G2/M phase. When *TP73* was knocked down, cells in the G1 phase decreased and cells in the S phase increased. This indicates that PLAGL2 may induce cell cycle arrest by regulating *TP73* to affect cell cycle inhibitors [149].

In addition, other studies have shown that p53-induced RING-H2, Pirh2, formed a dimer through its N-terminus and C-terminus [150]. PLAGL2 can interact with Pirh2 dimers, which can inhibit the degradation of Pirh2 mediated by the proteasome. *Pirh2* is the target gene of p53 and is up-regulated by p53, but the Pirh2 protein can negatively regulate the level and stability of p53 [150]. Experiments have shown that after silencing *Pirh2*, it will cause an increase in p53 levels, an increase in the portion of cells in the G1 phase, and a decrease in the S phase. Therefore, PLAGL2 may regulate p53 by interacting with Pirh2 and indirectly participate in the regulation of the cell cycle [150].

## 3. Another Seven N-Myc Target Genes

There are another seven N-Myc target genes according to the initial screening. These target genes have obvious effects on cell cycle regulation and tumor cell proliferation, which also have the potential to serve as targets for anti-tumor drugs. In order to facilitate reading and understanding, we summarized the regulations and mechanisms of these target genes in Table 1. However, the specific regulatory mechanism in the cell is currently unclear, and more in-depth research is needed in the future.

### 3.1. GLDC

Glycine decarboxylase (*GLDC*) is an environment-dependent metabolic oncogene. *GLDC* drives the occurrence of non-small cell lung cancer (NSCLC) and regulates the proliferation of cancer cells by promoting pyrimidine biosynthesis, glycolysis, and sarcosine production [174]. In gastric cancer, *GLDC* acts as a tumor suppressor gene, and the hypermethylation of its promoter makes it silent at the transcriptional level and promotes the occurrence of gastric cancer [175].

In *MYCN*-amplified NB cell lines, *MYCN* overexpression significantly increased the expression of *GLDC* [157]. Sequence detection and ChIP-qPCR revealed that N-Myc bound to the E-boxes in the *GLDC* promoter region and the first intron [157]. In summary, *MYCN* regulates the expression of *GLDC* in NB at the transcriptional level.

Knockdown of *GLDC* caused cell proliferation inhibition and G1 phase arrest [157]. Experiments have shown that down-regulation of the *GLDC* gene led to a significant reduction in the mRNA expression of cyclins and CDKs, including *CCNA2*, *CCNB1*, *CDK1*, *CDK2*, *CCND1*, *CCNE1*, DNA polymerase epsilon 2 (*POLE2*), and minichromosome maintenance complex component 5 (*MCM5*), which induced G1 blockade and inhibited cell proliferation [157]. It also caused changes in a variety of metabolic pathways, among which a significant reduction in purine and cholesterol synthesis can decrease the expression of cyclins and CDKs and inhibit cell proliferation [157].

GLDC is the P protein in the glycine cleavage system and can be combined with glycine to transfer the methylamine group of glycine to the T protein [158]. GLDC participates in the first and rate-limiting step of glycine decomposition [157]. It catalyzes the conversion of glycine into carbon dioxide, ammonia, and 5,10-methylenetetrahydrofolate (CH2-THF) [159]. However, CH2-THF drives the new synthesis of thymine and the biosynthesis of pyrimidine, thereby regulating the synthesis of nucleotides during cell proliferation [160].

### 3.2. TERT

Telomerase reverse transcriptase (TERT) is a component of telomerase. Compared with most normal cells lacking telomerase activity, telomerase activity is up-regulated in many malignant tumors, including thyroid cancer, NB, etc., allowing cancer cells to replicate indefinitely [4,176]. Evidence shows that TERT can prevent cell cycle arrest and prevent cell apoptosis induced by poor culture conditions in vitro [177,178]. 

In NB, N-Myc binds to the typical E-box near the transcription start site of the *TERT* gene, and the up-regulation of *MYCN* is accompanied by a 10- to 20-fold increase in *TERT* expression, suggesting that *TERT* is a target gene of N-Myc [88].

In Burkitt’s lymphoma, cells treated with BIBR (TERT inhibitor) showed changes in the cell cycle spectrum, with decreased cells in the G1 phase, the disappearance of cells in the G2/M phase, and a large accumulation of cells in the S phase. The inhibition of TERT leads to DNA damage, then ataxia-telangiectasia mutated (ATM) and ATM and Rad3-related (ATR) kinases are phosphorylated after sensing DNA-damage response (DDR) signals, thus activating checkpoint kinase 1 (Chk1), checkpoint kinase 2 (Chk2), and p53 proteins, and further phosphorylating Cdc25A protein to induce cell cycle arrest and accumulation in the S phase [151].

In many cancer cell lines, the decrease in *hTERT* (human TERT) expression is related to the significant down-regulation of *CCND1*, and the high expression of *hTERT* significantly up-regulates the expression of *CCND1*. In addition, the transcriptional activity and nuclear localization of Cyclin D1 are also regulated by TRET, suggesting that TERT promotes cell cycle progression by regulating the key cell cycle regulator Cyclin D1 [152].

### 3.3. PBK

The PDZ-binding kinase/T-LAK-cell-originated protein kinase (PBK/TOPK) is a serine-threonine kinase involved in the formation of the spindle and the process of mitosis [171]. *PBK* is highly expressed in lymphoma, myeloma, and primary hematological tumors, which is closely related to the malignant potential of these tumors [179,180,181].

In NB, the expression of *MYCN* and *PBK* is positively correlated. Knocking down *MYCN* leads to a decrease in *PBK* mRNA and protein levels. Chromatin immunoprecipitation sequencing (ChIP-seq) data analysis showed that N-Myc bound to the *PBK* promoter and was accompanied by the acetylation of lysine 27 of histone H3 (H3K27Ac), indicating that N-Myc can promote the transcription process of *PBK* [168].

The expression of *PBK* is related to mitosis. During mitosis, PBK forms a complex with Cyclin B1-CDK1 and phosphorylates the protein regulator of cytokinesis 1 (PRC1) in a Cyclin B1-CDK1-dependent manner. PRC1 is a microtubule-binding protein that participates in the formation of the mitotic spindle and promotes cytokinesis [171].

In human colon cancer cells, PBK interacts with p53, which contains the DNA binding domain (DBD), thereby down-regulating the transactivation function of p53 [169]. The CKI *p21* is a well-known transcription target of p53 and plays a crucial role in mediating growth arrest [172]. Upon silencing *PBK*, the promoter activity of *p21* and its mRNA significantly increased. Experiments proved that the overexpression of PBK down-regulated the expression of *p21* by reducing the recruitment of p53 to the *p21* promoter [169]. After knocking out *PBK*, the number of cells in the G2/M phase increased significantly and cell growth slowed down, indicating that the knockdown of the *PBK* gene may lead to cell cycle arrest and inhibit cell proliferation [169]. In addition, the expression levels of many other p53 target genes are also significantly changed due to *PBK* gene knockdown, such as the G2/M phase-specific E3 ubiquitin-protein ligase (*G2E3*) [170], Dual specificity protein phosphatase 1 (*DUSP1*) [182], etc., which regulate cell proliferation by participating in cell cycle regulation [169].

### 3.4. SGO1

Shugoshin 1 (SGO1) is an important protective protein for centromeric condensation [133]. Sgo1 is expressed during proliferation and must be located in the inner centromere in order to exert its cohesion protection function [133,183,184,185]. Sgo1 deficiency causes damage to the spindle checkpoint, resulting in incorrect separation of sister chromatids, leading to chromosomal instability (CIN) and tumor transformation [163,186,187,188].

In the *SGO1* genome sequence, there are four E-boxes (1–4) in the 4kb region upstream of the start codon of *SGO1*, and an E-box is located in the intron of *SGO1* [161]. ChIP analysis showed that N-Myc was recruited in E-box1 and E-box2 upstream of *SGO1*. Overexpression of *MYCN* induced up-regulation of *SGO1* expression [161]. In addition, when *MYCN* was down-regulated in IMR32 cells, Sgo1 protein levels decreased. These results indicate that N-Myc up-regulates the expression of *SGO1* by combining with E-box1 and E-box2 [161].

In NB cells overexpressing *MYCN*, using shRNA to knock down *SGO1* severely inhibited cell proliferation, induced DNA damage, and caused cells to accumulate in the G2/M phase. [161]

In prostate cancer cells, the high expression of *SGO1* promotes cell proliferation and cell cycle progression [162]. Flow cytometry results showed that If *SGO1* was induced to overexpress, the ratio of G0/G1 phase in cells decreased, and the ratio of S and G2/M phase increased [162]. When *SGO1* was knocked down, the ratio of the G0/G1 phase increased, and the ratio of the S phase and the G2/M phase decreased. At the same time, Western Blot experiments showed that the expression of Cyclin A, CDK2, and Cyclin D1 was significantly reduced [162].

Among lung cancer cells, overexpression or knockdown of shugoshin-like 1 (*SGOL1*) prevents the correct alignment of the chromosomes in the metaphase equatorial plate, resulting in a delay at the beginning of the anaphase of mitosis [163].

### 3.5. AURKB

The serine/threonine protein kinase AURKB is the catalytic subunit of the chromosome passenger complex. It regulates many aspects of mitosis, including spindle checkpoints, chromosome segregation, and cytokinesis [189]. Overexpression of *AURKB* in a variety of human cancers shows a tendency for high malignancy and is positively correlated with poor prognosis [190].

ChIP-Seq data indicated that N-Myc can bind to E-box in the promoter region of *AURKB* [191]. GO analysis showed that the induction of high levels of *MYCN* resulted in the up-regulation of *AURKB* expression [153].

AURKB plays a regulatory role in the transition from the G2 phase to cytokinesis [190]. AURKB moves along the kinetochore to the equatorial region after the beginning of the later period, which is necessary for chromosome separation and cytokinesis [192]. Experiments showed that the lack of Aurora B interfered with the cell cycle and prevented the cell from completing cytokinesis, thus forming a tetraploid with two centrosomes [190]. After knocking out *AURKB*, the phosphorylation of histone H3 was significantly reduced, causing chromosome aggregation obstacles [155]. After injection of the AURKB antibody, AURKB is inhibited, blocking chromosome separation, covering the spindle checkpoint, and disrupting microtubule dynamics in mitosis [156].

In clear cell renal cell carcinoma, inhibition of AURKB induced cell accumulation in the G2/M phase and led to the down-regulation of Cyclin B and Cdc2 [154]. It was found that the expression of Cdc25C decreased and the expression of p-Cdc2 (Tyr15) increased in the cells that silenced Aurora kinase. The accumulation of p-Cdc2 kept Cdc2 in an inactive state, causing the cells to be blocked in the G2/M phase [154].

### 3.6. E2F5

E2F transcription factor 5 (E2F5) is a member of the E2F family and plays an important role in regulating the proliferation of many types of tumors [164,193,194]. E2F5 can bind to pocket protein p107 or p130, inhibit tumor growth, and block cell cycle progression in the G1 phase [166,167]. 

ChIP experiments confirmed that N-Myc can directly bind to the E-box in the *E2F5* gene promoter and up-regulate its expression [164]. Tetracycline induced the SHEP-Tet21N cell line to up-regulate the expression of *MYCN*, and Western Blot showed that the level of E2F5 protein increased [164]. 

A CCK-8 experiment found that the down-regulation of *E2F5* significantly inhibited the proliferation of NB cells. When *E2F5* was down-regulated by siRNA transfection, the proportion of cells in the G0/G1 phase increased, and the expression levels of CDK2 and CDK6 decreased [164]. It is suggested that E2F5 regulates the cell cycle progression of NB by affecting CDK2 and CDK6 [164]. In glioblastoma multiforme (GBM), silencing *E2F5* effectively inhibited the proliferation of GBM cells, and the cell cycle was arrested in the G0/G1 phase [165]

### 3.7. TEAD4

The TEA domain transcription factor 4 (TEAD4), also known as transcriptional enhancer factor-3 (TEF-3), is a key molecule in the TEAD family. *TEAD4* is up-regulated in a variety of tumors and is a potential tumor prognostic marker, especially in *MYCN*-amplified NB cells, and is a key component to drive their proliferation [173,195,196]. 

Studies have confirmed that N-Myc binds to the *TEAD4* promoter region to up-regulate its expression. Silencing *MYCN* can down-regulate *TEAD4* at the protein level. In addition, TEAD4 binds to the *MYCN* promoter. Therefore, they have a positive feedback loop [173].

In NB, silencing the *TEAD4* gene induces significant aggregation of cells in the G0/G1 phase and a decrease in cells in the S phase. At the genetic level, multiple genes involved in cell cycle progression and DNA replication are inhibited, including cyclin-dependent kinases (*CDK2*, *CDK1*, *CDC25B*), cyclins (*CCND1*), DNA replication proliferating cell nuclear antigen (*PCNA*), minichromosome maintenance complex component 7 (*MCM7*), Cdc6), checkpoint kinases (*CHEK1*, *CHEK2*, *WEE1*), and others. The regulation of these genes by *TEAD4* is not affected by TAZ/YAP in the Hippo classic pathway [173]. 

## 4. Discussion

Neuroblastoma is one of the serious threats to the life and health of children, especially infants and young children [197]. Because its onset is insidious and lacks specificity, it is difficult to detect and diagnose at an early stage, and it has often metastasized by the time it is diagnosed and the overall degree of malignancy is relatively high [198,199]. The risk classification of neuroblastoma is based on a comprehensive assessment of the child’s age, *MYCN* gene amplification, and 11q chromosome aberrations [200,201]. Among them, the amplification and high expression of the *MYCN* gene are often positively correlated with the malignant progression and poor prognosis of various malignant tumors such as NB [202,203], receiving widespread concern. 

In addition to the target genes mentioned in the text, the N-Myc downstream-regulated gene (*NDRG*) is a classic N-Myc downstream target gene (including *NDRG1-4*), and its expression is inhibited by N-Myc in NB [204]. The expression of *NDRG1* is biphasic throughout the cell cycle, peaking in the G1 and G2/M phases and reduced to the lowest level in the S phase, indicating that it may play a potential role in the G0/G1 block by changing the expression of *p21*^Waf1/Cip1^ and *CDK1/4* [205]. However, although NDRG1 can up-regulate *p21*^Waf1/Cip1^ in prostate and lung tumor cells, it does not affect the cell cycle and proliferation but instead inhibits cell migration [206]. However, the overexpression of *NDRG1* has also been confirmed to reduce the expression of the Wnt response gene *CCND1*, thereby inhibiting the process of the cell cycle [207]. In addition, studies have shown that NDRG1 may be related to the attachment of mitotic spindles during shedding and the regulation of cytokinesis [208]. All of this evidence indicates that NDRG1 is bidirectional and cell-type-dependent in regulating the cell cycle, suggesting that it has multiple limitations as a target of anti-tumor drugs.

As for the gene of the Mre11/Rad50/NBS1 (MRN) complex, studies have shown that *RAD50* is the target gene of N-Myc. Although there is no evidence that the other two are direct targets of N-Myc, both of them contain a CACGTG MYC binding sequence E-box, making them potential target genes of N-Myc [209]. MRN is the main sensor of a DNA double-strand break (DSB) [210]. When DNA damage is detected, it activates signaling molecules, such as protein kinase ATM, to trigger a wide range of DNA damage responses, including cell cycle arrest [211]. Studies showed that the knockdown of *NBN* or inhibition of Mre11 can effectively inhibit the proliferation of *MYCN*-amplified cells [209]. In summary, MRN as a potential downstream target of N-Myc may play a role in inhibiting tumor proliferation and could be used as a target for anti-tumor drugs, but studies on this have been inadequate.

This article summarizes and explains the mechanism by which N-Myc promotes tumor cell proliferation by regulating the cell cycle and cell division and provides new ideas for research on targeted drugs. At present, the research and development of targeted drugs for *MYCN* primarily has the following ideas: (1) Prevent the abnormal expression of *MYCN*: For example, JQ1, a small-molecule inhibitor of the bromodomain and the extraterminal domain (BET) protein, can effectively down-regulate *MYCN* gene transcription and inhibit the proliferation of *MYCN*-amplified NB cells [212]. In addition, studies have shown that the inhibitors of CDK7(THZ1) or CDK9(CYC065) can disrupt abnormal *MYCN*-driven transcription and inhibit *MYCN* gene transcription, becoming potential anti-cancer drugs [213,214]. (2) Furthermore, we can promote the degradation of N-Myc by acting on the stability regulation network of N-Myc [215]. For example, PLK1 can counteract the F-box and WD domain protein 7 (FBXW7)-mediated degradation of N-Myc by destroying the stability of FBXW7 ubiquitin ligase complex, thereby increasing the stability of N-Myc [122]. PLK1 inhibitor BI 2356 shows strong anti-tumor activity in NB cells in vitro and in vivo [216]. Moreover, in NB and SCLC, *MYCN*-amplified tumor cells are more sensitive to PLK1 inhibitor treatment than tumors with normal N-Myc copy numbers [122]. In addition, studies have shown that N-Myc may be methylated by PRMT5 to reduce its degradation by the proteasome [217]. Treatment with PRMT5 inhibitor EPZ015666 resulted in decreased MYC protein levels and medulloblastoma cell growth, indicating that PRMT5 inhibitors are potential treatments for *MYCN*-driven cancer [218]. (3) The formation of heterodimers between N-Myc and MAX could be inhibited, thereby inhibiting the effect of N-Myc on target genes [219]. The compound can compete with MAX for the binding site HLH-Zip on N-Myc, such as 10058-F4 [220] and MYCi361 [221], or reduce the formation of the MAX- N-Myc complex by stabilizing the MAX homodimer [222]. (4) Lastly, the target gene of N-Myc proteins could be inhibited, such as MDM2 [223]. All the small-molecule drugs mentioned above are summarized and sorted into Table 2, which will provide more information about compounds including the corresponding structure, target, cell-free assay, cell data, and clinical trials.

In this paper, we summarized ten target genes of N-Myc and their regulatory networks for the cell cycle (primarily Cyclins and CDK, but also involved in proteins that play key regulatory roles in cell cycle progression, including proteins at cell cycle checkpoints). However, at the same time, we also noted that another seven N-Myc target genes showed abnormal expression in neuroblastoma, and the abnormal expression (overexpression or inhibition) of these genes led to the abnormal cycle of various tumor cells, suggesting the existence of a potential network of action, which requires further study. 

Moreover, given that MYNC amplification is a high-risk factor for many tumors, MYCN affects cell cycle progression by targeting downstream genes, and many antitumor drugs targeting MYCN downstream genes have been developed with great success, we believe that MYCN can also be used as a target for antitumor drug development, and has great research potential and application value.

**Table 2:** Small-molecule compounds targeting N-MYC and its downstream.

General Mode of Action	Compound	Structure	Target	Cell-Free Assay	Cell Data	Clinical Trails
Prevent the abnormal expression of MYCN	(+)-JQ1	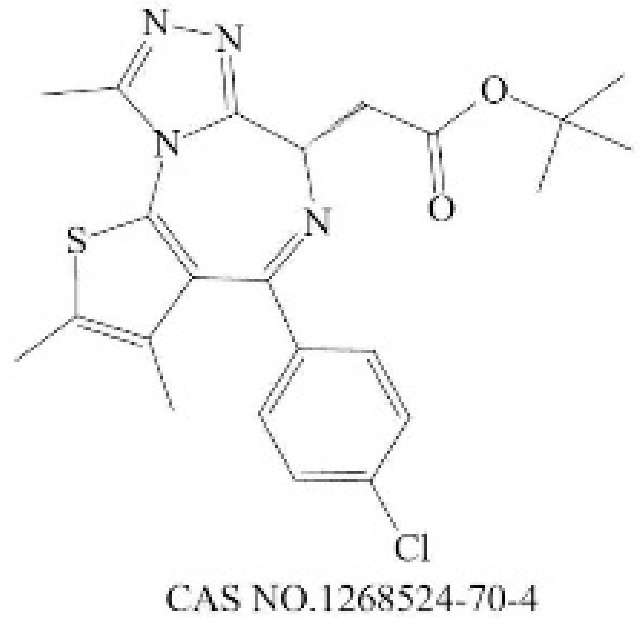	BRD4(1/2) [224]	77/33 (1 h) [224]	4 (NMC 11060, 72 h) [224]	N
	THZ1	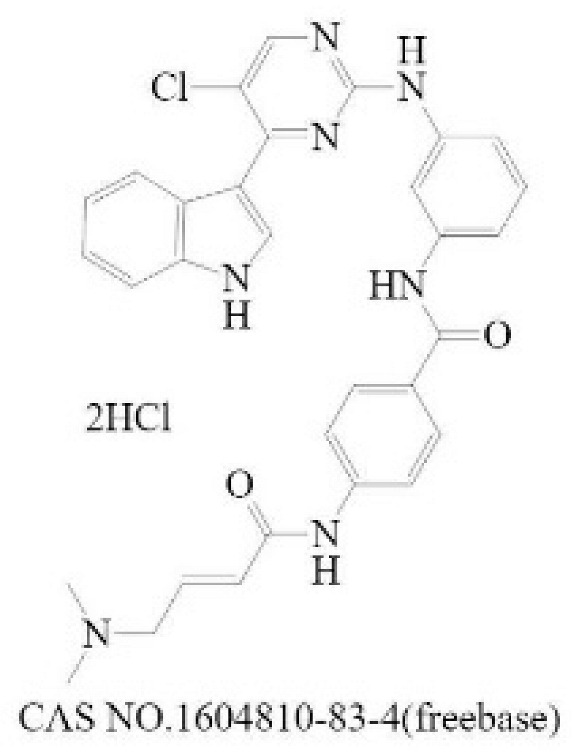	CDK7 [225]	3.2 (3 h) [225]	50 (Jurkat cells, 72 h) [226]	N
	CYC065	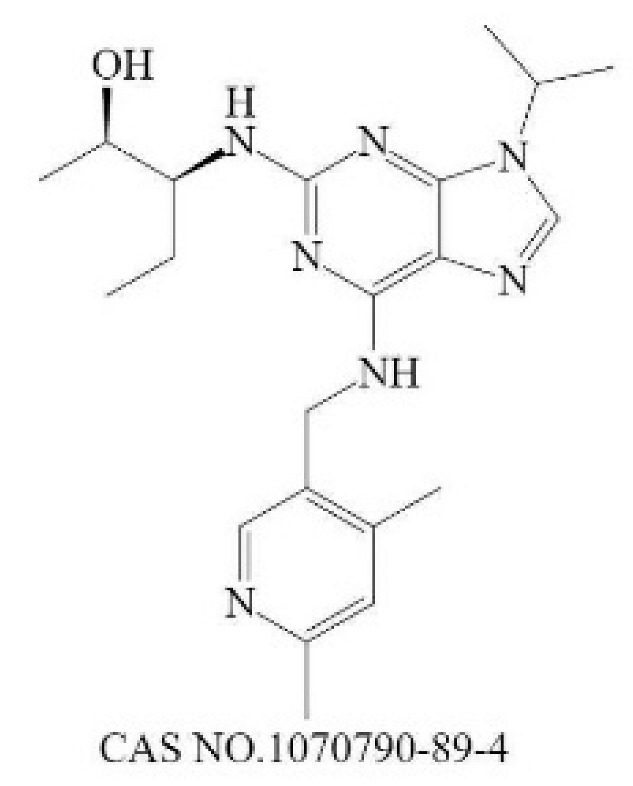	CDK9 [227]	26 ^a^ [227]	370 (Hop63 24 h) [228]	Phase 1/2: Solid Tumor, Adult Lymphoma (Recruiting) [229]
Promote the degradation of N-Myc	EPZ015666	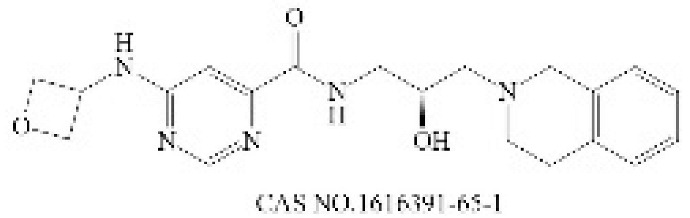	PRMT5 [230]	22 (120 h) [231]	96 (Z-138, 12 days) [231]	N
	BI 2536	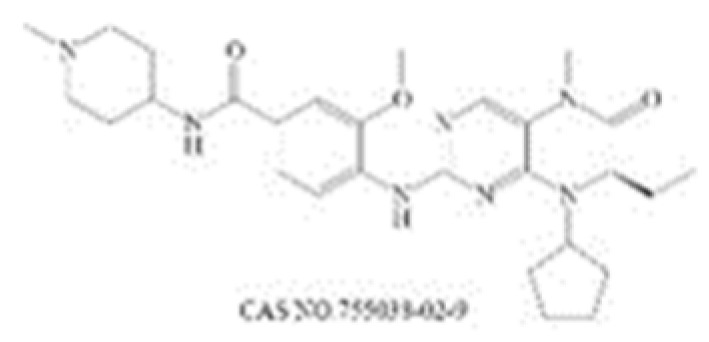	PLK1 [232]	0.83 (45 min) [232]	1.78 (NALM-6, 72 h) [233]	Phase 1: NSCLC; advanced solid tumours; Pancreatic Neoplasms; Non-Hodgkin’s Lymphoma Phase 2: AML; Prostatic Neoplasms; NSCLC; SCLC; Pancreatic Cancer; Breast Cancer/Endometrial Cancer/Head and Neck Cancer/Melanoma (Skin)/Ovarian Cancer/Sarcoma [234]
Inhibit the formation of heterodimers between N-Myc and MAX	MYCi361	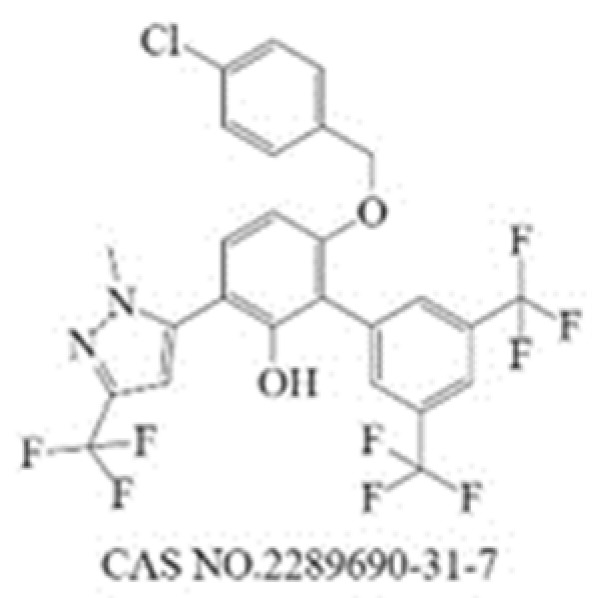	MYC [221]	3200 (Kd) [221]	490 (SK-NB2, 120 h) [221]	N
Inhibit downstream target of N-Myc	Nutlin-3	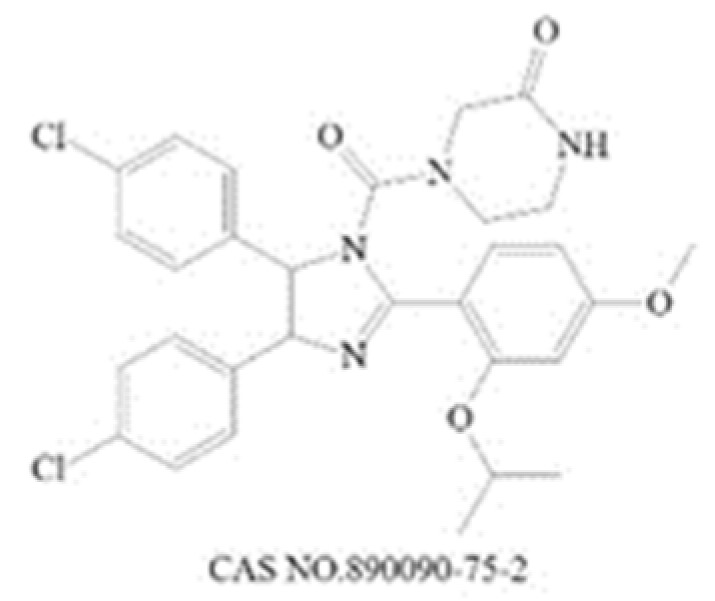	Mdm2 [235]	90 (1 h) [236]	650 (U87MG,48 h) [237]	N

^a^ The time of the experiment is not indicated in the literature.

## Figures and Tables

**Table 1:** Target genes up-regulated by N-Myc and their regulating targets. The seven target genes listed in the form show their ability to regulate the process of cell cycle in a relatively unspecific way. Up- or down-regulation of the expression of these MYCN target genes affects the expression of genes further downstream, but there is no direct evidence of the regulatory mechanism.

Target Gene	Phase	Regulating Target
*TERT* [88]	S [151]	Inhibition of TERT leads to DDR and down-regulation of *CCND1*. The high expression of *TERT* significantly up-regulated the expression of *CCND1* [151,152].
*AURKB* [153]	G2/M [154], M [155,156]	Inhibition of AURKB results in down-regulation of *CCNB* and *CDC2* and *CDC25C*, significantly reduces phosphorylation of histone H3, and blocks chromosome segregation and cytokinesis [154,155,156].
*GLDC* [157]	G1 [157]	The expressions of *CCNA2*, *CCNB1*, *CDK1*, *CDK2*, *CCND1*, *CCNE1*, *POLE2* and *MCM5* are decreased after knocking down *GLDC*. GLDC can also regulate the synthesis of nucleotides and cholesterol [157,158,159,160].
*SGO1* [161]	G0/G1 [162], S [162], G2/M [161,162]	The expressions of *CCNA*, *CDK2*, *CCND1* are decreased after knocking down *SGO1*. SGO1 regulate separation of sister chromatids [161,162,163].
*E2F5* [164]	G0/G1 [164,165], G1 [166,167]	The expressions of *CDK2*, *CDK6* are decreased after knocking down *E2F5* [164].
*PBK* [168]	G2/M [169,170], M [171]	PBK forms a complex with Cyclin B1-CDK1 and phosphorylates PRC1 in a Cyclin B1-CDK1-dependent manner, thereby participating in the formation of the mitotic spindle and promotes cytokinesis [171]. PBK interacts with p53 containing the DBD domain to down-regulate the transactivation function of *TP53*. Over-expression of *PBK* down-regulates the expression of *p21* by reducing the recruitment of *p21* promoter to p53 [169,170,172].
*TEAD4* [173]	G0/G1 [173]	After silencing the *TEAD4* gene, cyclin-dependent kinases (*CDK2*, *CDK1*, *CDC25B*), cyclins (*CCND1*), DNA replication proliferating cell nuclear antigen (*PCNA*), minichromosome maintenance complex component 7 (*MCM7*), Cdc6, checkpoint kinases (*CHEK1*, *CHEK2*, *WEE1*) and other proteins are inhibited [173].

## Data Availability

All relevant data and material are available for any inquiry.

## Supplementary Materials

The following supporting information can be downloaded at: https://www.mdpi.com/article/10.3390/molecules28031141/s1, Table S1. The summarization of 10 target genes of N-myc in Section 2. These target genes regulated by N-Myc and further affecting the different process of cell cycle in multiple ways.

## References

[1] Ward E., DeSantis C., Robbins A., Kohler B., Jemal A. (2014). Childhood and adolescent cancer statistics, 2014. CA Cancer J. Clin..

[2] Tomioka N., Oba S., Ohira M., Misra A., Fridlyand J., Ishii S., Nakamura Y., Isogai E., Hirata T., Yoshida Y. (2008). Novel risk stratification of patients with neuroblastoma by genomic signature, which is independent of molecular signature. Oncogene.

[3] Cheung N.K., Zhang J., Lu C., Parker M., Bahrami A., Tickoo S.K., Heguy A., Pappo A.S., Federico S., Dalton J. (2012). Association of age at diagnosis and genetic mutations in patients with neuroblastoma. JAMA.

[4] Peifer M., Hertwig F., Roels F., Dreidax D., Gartlgruber M., Menon R., Krämer A., Roncaioli J.L., Sand F., Heuckmann J.M. (2015). Telomerase activation by genomic rearrangements in high-risk neuroblastoma. Nature.

[5] Campbell K., Gastier-Foster J.M., Mann M., Naranjo A.H., van Ryn C., Bagatell R., Matthay K.K., London W.B., Irwin M.S., Shimada H. (2017). Association of MYCN copy number with clinical features, tumor biology, and outcomes in neuroblastoma: A report from the Children’s Oncology Group. Cancer.

[6] Marrano P., Irwin M.S., Thorner P.S. (2017). Heterogeneity of MYCN amplification in neuroblastoma at diagnosis, treatment, relapse, and metastasis. Genes Chromosomes Cancer.

[7] Duffy M.J., O’Grady S., Tang M., Crown J. (2021). MYC as a target for cancer treatment. Cancer Treat. Rev..

[8] Beaulieu M.E., Castillo F., Soucek L. (2020). Structural and Biophysical Insights into the Function of the Intrinsically Disordered Myc Oncoprotein. Cells.

[9] Carroll P.A., Freie B.W., Mathsyaraja H., Eisenman R.N. (2018). The MYC transcription factor network: Balancing metabolism, proliferation and oncogenesis. Front. Med..

[10] Beltran H. (2014). The N-myc Oncogene: Maximizing its Targets, Regulation, and Therapeutic Potential. Mol. Cancer Res..

[11] Liu Z., Chen S.S., Clarke S., Veschi V., Thiele C.J. (2020). Targeting MYCN in Pediatric and Adult Cancers. Front. Oncol..

[12] Wenzel A., Schwab M. (1995). The mycN/max protein complex in neuroblastoma. Short review. Eur. J. Cancer.

[13] Murphy D.M., Buckley P.G., Bryan K., Das S., Alcock L., Foley N.H., Prenter S., Bray I., Watters K.M., Higgins D. (2009). Global MYCN transcription factor binding analysis in neuroblastoma reveals association with distinct E-box motifs and regions of DNA hypermethylation. PLoS ONE.

[14] Sun Y., Bell J.L., Carter D., Gherardi S., Poulos R.C., Milazzo G., Wong J.W., Al-Awar R., Tee A.E., Liu P.Y. (2015). WDR5 Supports an N-Myc Transcriptional Complex That Drives a Protumorigenic Gene Expression Signature in Neuroblastoma. Cancer Res..

[15] Yang J., AlTahan A.M., Hu D., Wang Y., Cheng P.H., Morton C.L., Qu C., Nathwani A.C., Shohet J.M., Fotsis T. (2015). The role of histone demethylase KDM4B in Myc signaling in neuroblastoma. J. Natl. Cancer Inst..

[16] Amente S., Milazzo G., Sorrentino M.C., Ambrosio S., di Palo G., Lania L., Perini G., Majello B. (2015). Lysine-specific demethylase (LSD1/KDM1A) and MYCN cooperatively repress tumor suppressor genes in neuroblastoma. Oncotarget.

[17] Zeid R., Lawlor M.A., Poon E., Reyes J.M., Fulciniti M., Lopez M.A., Scott T.G., Nabet B., Erb M.A., Winter G.E. (2018). Enhancer invasion shapes MYCN-dependent transcriptional amplification in neuroblastoma. Nat. Genet..

[18] Agarwal S., Milazzo G., Rajapakshe K., Bernardi R., Chen Z., Barbieri E., Koster J., Perini G., Coarfa C., Shohet J.M. (2018). MYCN acts as a direct co-regulator of p53 in MYCN amplified neuroblastoma. Oncotarget.

[19] O’Brien E.M., Selfe J.L., Martins A.S., Walters Z.S., Shipley J.M. (2018). The long non-coding RNA MYCNOS-01 regulates MYCN protein levels and affects growth of MYCN-amplified rhabdomyosarcoma and neuroblastoma cells. BMC Cancer.

[20] Tchakarska G., Sola B. (2020). The double dealing of cyclin D1. Cell Cycle.

[21] Tatum N.J., Endicott J.A. (2020). Chatterboxes: The structural and functional diversity of cyclins. Semin. Cell Dev. Biol..

[22] Malumbres M. (2014). Cyclin-dependent kinases. Genome Biol..

[23] Dang F., Nie L., Wei W. (2021). Ubiquitin signaling in cell cycle control and tumorigenesis. Cell Death Differ..

[24] Mercatelli D., Balboni N., Palma A., Aleo E., Sanna P.P., Perini G., Giorgi F.M. (2021). Single-Cell Gene Network Analysis and Transcriptional Landscape of MYCN-Amplified Neuroblastoma Cell Lines. Biomolecules.

[25] Takahashi K., Tanabe K., Ohnuki M., Narita M., Ichisaka T., Tomoda K., Yamanaka S. (2007). Induction of pluripotent stem cells from adult human fibroblasts by defined factors. Cell.

[26] Li Z., Li X., Li C., Su Y., Fang W., Zhong C., Ji W., Zhang Q., Su C. (2014). Transcription factor OCT4 promotes cell cycle progression by regulating CCND1 expression in esophageal carcinoma. Cancer Lett..

[27] Schoenhals M., Kassambara A., de Vos J., Hose D., Moreaux J., Klein B. (2009). Embryonic stem cell markers expression in cancers. Biochem. Biophys. Res. Commun..

[28] Suenaga Y., Nakatani K., Nakagawara A. (2020). De novo evolved gene product NCYM in the pathogenesis and clinical outcome of human neuroblastomas and other cancers. Jpn. J. Clin. Oncol..

[29] Yang L., Zheng J., Xu T., Xiao X. (2013). Downregulation of OCT4 promotes differentiation and inhibits growth of BE (2)-C human neuroblastoma I-type cells. Oncol. Rep..

[30] Su C. (2016). Survivin in survival of hepatocellular carcinoma. Cancer Lett..

[31] Bai M., Yuan M., Liao H., Chen J., Xie B., Yan D., Xi X., Xu X., Zhang Z., Feng Y. (2015). OCT4 pseudogene 5 upregulates OCT4 expression to promote proliferation by competing with miR-145 in endometrial carcinoma. Oncol. Rep..

[32] Han S.M., Han S.H., Coh Y.R., Jang G., Chan Ra J., Kang S.K., Lee H.W., Youn H.Y. (2014). Enhanced proliferation and differentiation of Oct4- and Sox2-overexpressing human adipose tissue mesenchymal stem cells. Exp. Mol. Med..

[33] Card D.A., Hebbar P.B., Li L., Trotter K.W., Komatsu Y., Mishina Y., Archer T.K. (2008). Oct4/Sox2-regulated miR-302 targets cyclin D1 in human embryonic stem cells. Mol. Cell. Biol..

[34] Lin S.L., Chang D.C., Ying S.Y., Leu D., Wu D.T. (2010). MicroRNA miR-302 inhibits the tumorigenecity of human pluripotent stem cells by coordinate suppression of the CDK2 and CDK4/6 cell cycle pathways. Cancer Res..

[35] Schoeftner S., Scarola M., Comisso E., Schneider C., Benetti R. (2013). An Oct4-pRb axis, controlled by MiR-335, integrates stem cell self-renewal and cell cycle control. Stem Cells.

[36] Fang Z.J., Lin M., Li C.X., Liu H., Gong C.J. (2020). A comprehensive review of the roles of E2F1 in colon cancer. Am. J. Cancer Res..

[37] Pennycook B.R., Barr A.R. (2020). Restriction point regulation at the crossroads between quiescence and cell proliferation. FEBS Lett..

[38] She S., Wei Q., Kang B., Wang Y.J. (2017). Cell cycle and pluripotency: Convergence on octamer-binding transcription factor 4 (Review). Mol. Med. Rep..

[39] Wierstra I., Alves J. (2006). Transcription and pluripotency: Convergence on octamer-binding transcrip factor FOXM1c is repressed by RB and activated by cyclin D1/Cdk4. Biol. Chem..

[40] Zhao R., Deibler R.W., Lerou P.H., Ballabeni A., Heffner G.C., Cahan P., Unternaehrer J.J., Kirschner M.W., Daley G.Q. (2014). A nontranscriptional role for Oct4 in the regulation of mitotic entry. Proc. Natl. Acad. Sci. USA.

[41] Yu K.R., Yang S.R., Jung J.W., Kim H., Ko K., Han D.W., Park S.B., Choi S.W., Kang S.K., Scholer H. (2012). CD49f enhances multipotency and maintains stemness through the direct regulation of OCT4 and SOX2. Stem Cells.

[42] Tsai C.C., Su P.F., Huang Y.F., Yew T.L., Hung S.C. (2012). Oct4 and Nanog Directly Regulate Dnmt1 to Maintain Self-Renewal and Undifferentiated State in Mesenchymal Stem Cells. Mol. Cell.

[43] Bedford M.T., Clarke S.G. (2009). Protein arginine methylation in mammals: Who, what, and why. Mol. Cell.

[44] Song C., Chen T., He L., Ma N., Li J.A., Rong Y.F., Fang Y., Liu M., Xie D., Lou W. (2020). PRMT1 promotes pancreatic cancer growth and predicts poor prognosis. Cell. Oncol..

[45] Liu L.M., Sun W.Z., Fan X.Z., Xu Y.L., Cheng M.B., Zhang Y. (2019). Methylation of C/EBPα by PRMT1 Inhibits Its Tumor-Suppressive Function in Breast Cancer. Cancer Res..

[46] Chuang C.Y., Chang C.P., Lee Y.J., Lin W.L., Chang W.W., Wu J.S., Cheng Y.W., Lee H., Li C. (2017). PRMT1 expression is elevated in head and neck cancer and inhibition of protein arginine methylation by adenosine dialdehyde or PRMT1 knockdown downregulates proliferation and migration of oral cancer cells. Oncol. Rep..

[47] Valentijn L.J., Koster J., Haneveld F., Aissa R.A., van Sluis P., Broekmans M.E., Molenaar J.J., van Nes J., Versteeg R. (2012). Functional MYCN signature predicts outcome of neuroblastoma irrespective of MYCN amplification. Proc. Natl. Acad. Sci. USA.

[48] Eberhardt A., Hansen J.N., Koster J., Lotta L.T., Wang S., Livingstone E., Qian K., Valentijn L.J., Zheng Y.G., Schor N.F. (2016). Protein arginine methyltransferase 1 is a novel regulator of MYCN in neuroblastoma. Oncotarget.

[49] Hua Z.Y., Hansen J.N., He M., Dai S.K., Choi Y., Fulton M.D., Lloyd S.M., Szemes M., Sen J., Ding H.F. (2020). PRMT1 promotes neuroblastoma cell survival through ATF5. Oncogenesis.

[50] Klerkx E.P., Lazo P.A., Askjaer P. (2009). Emerging biological functions of the vaccinia-related kinase (VRK) family. Histol. Histopathol..

[51] Huang W., Cui X., Chen Y., Shao M., Shao X., Shen Y., Liu Q., Wu M., Liu J., Ni W. (2016). High VRK1 expression contributes to cell proliferation and survival in hepatocellular carcinoma. Pathol. Res. Pract..

[52] Ben Z., Gong L., Qiu Y. (2018). High expression of VRK1 is related to poor prognosis in glioma. Pathol. Res. Pract..

[53] Colmenero-Repiso A., Gómez-Muñoz M.A., Rodríguez-Prieto I., Amador-Álvarez A., Henrich K.O., Pascual-Vaca D., Okonechnikov K., Rivas E., Westermann F., Pardal R. (2020). Identification of VRK1 as a New Neuroblastoma Tumor Progression Marker Regulating Cell Proliferation. Cancers.

[54] Santos C.R., Rodríguez-Pinilla M., Vega F.M., Rodríguez-Peralto J.L., Blanco S., Sevilla A., Valbuena A., Hernández T., van Wijnen A.J., Li F. (2006). VRK1 signaling pathway in the context of the proliferation phenotype in head and neck squamous cell carcinoma. Mol. Cancer Res..

[55] Kang T.H., Park D.Y., Kim W., Kim K.T. (2008). VRK1 phosphorylates CREB and mediates CCND1 expression. J. Cell Sci..

[56] Lee N., Kwon J.H., Kim Y.B., Kim S.H., Park S.J., Xu W., Jung H.Y., Kim K.T., Wang H.J., Choi K.Y. (2015). Vaccinia-related kinase 1 promotes hepatocellular carcinoma by controlling the levels of cell cycle regulators associated with G1/S transition. Oncotarget.

[57] Valbuena A., Lopez-Sanchez I., Lazo P.A. (2008). Human VRK1 Is an Early Response Gene and Its Loss Causes a Block in Cell Cycle Progression. PLoS ONE.

[58] Ren Z., Geng J., Xiong C., Li X., Li Y., Li J., Liu H. (2020). Downregulation of VRK1 reduces the expression of BANF1 and suppresses the proliferative and migratory activity of esophageal cancer cells. Oncol. Lett..

[59] Jamin A., Wicklund A., Wiebe M.S. (2014). Cell- and virus-mediated regulation of the barrier-to-autointegration factor’s phosphorylation state controls its DNA binding, dimerization, subcellular localization, and antipoxviral activity. J. Virol..

[60] Nichols R.J., Wiebe M.S., Traktman P. (2006). The vaccinia-related kinases phosphorylate the N′ terminus of BAF, regulating its interaction with DNA and its retention in the nucleus. Mol. Biol. Cell.

[61] Aihara H., Nakagawa T., Mizusaki H., Yoneda M., Kato M., Doiguchi M., Imamura Y., Higashi M., Ikura T., Hayashi T. (2016). Histone H2A T120 Phosphorylation Promotes Oncogenic Transformation via Upregulation of Cyclin D1. Mol. Cell.

[62] Deng T., Yan G., Song X., Xie L., Zhou Y., Li J., Hu X., Li Z., Hu J., Zhang Y. (2018). Deubiquitylation and stabilization of p21 by USP11 is critical for cell-cycle progression and DNA damage responses. Proc. Natl. Acad. Sci. USA.

[63] Jia T., Zhang L., Duan Y., Zhang M., Wang G., Zhang J., Zhao Z. (2014). The differential susceptibilities of MCF-7 and MDA-MB-231 cells to the cytotoxic effects of curcumin are associated with the PI3K/Akt-SKP2-Cip/Kips pathway. Cancer Cell Int..

[64] Wu J., Su H.K., Yu Z.H., Xi S.Y., Guo C.C., Hu Z.Y., Qu Y., Cai H.P., Zhao Y.Y., Zhao H.F. (2020). Skp2 modulates proliferation, senescence and tumorigenesis of glioma. Cancer Cell Int..

[65] Li C., Du L., Ren Y., Liu X., Jiao Q., Cui D., Wen M., Wang C., Wei G., Wang Y. (2019). SKP2 promotes breast cancer tumorigenesis and radiation tolerance through PDCD4 ubiquitination. J. Exp. Clin. Cancer Res..

[66] Wei X., Li X., Yan W., Zhang X., Sun Y., Zhang F. (2018). SKP2 Promotes Hepatocellular Carcinoma Progression Through Nuclear AMPK-SKP2-CARM1 Signaling Transcriptionally Regulating Nutrient-Deprived Autophagy Induction. Cell. Physiol. Biochem..

[67] Evans L., Chen L., Milazzo G., Gherardi S., Perini G., Willmore E., Newell D.R., Tweddle D.A. (2015). SKP2 is a direct transcriptional target of MYCN and a potential therapeutic target in neuroblastoma. Cancer Lett..

[68] Muth D., Ghazaryan S., Eckerle I., Beckett E., Pöhler C., Batzler J., Beisel C., Gogolin S., Fischer M., Henrich K.O. (2010). Transcriptional repression of SKP2 is impaired in MYCN-amplified neuroblastoma. Cancer Res..

[69] Zhang L., Wang C. (2006). F-box protein Skp2: A novel transcriptional target of E2F. Oncogene.

[70] Binne U.K., Classon M.K., Dick F.A., Wei W., Rape M., Kaelin W.G., Naar A.M., Dyson N.J. (2007). Retinoblastoma protein and anaphase-promoting complex physically interact and functionally cooperate during cell-cycle exit. Nat. Cell Biol..

[71] Assoian R.K., Yung Y. (2008). A reciprocal relationship between Rb and Skp2—Implications for restriction point control, signal transduction to the cell cycle and cancer. Cell Cycle.

[72] Hydbring P., Castell A., Larsson L.G. (2017). MYC Modulation around the CDK2/p27/SKP2 Axis. Genes..

[73] Yung Y., Walker J.L., Roberts J.M., Assoian R.K. (2007). A Skp2 autoinduction loop and restriction point control. J. Cell Biol..

[74] Kumarasamy V., Vail P., Nambiar R., Witkiewicz A.K., Knudsen E.S. (2021). Functional Determinants of Cell Cycle Plasticity and Sensitivity to CDK4/6 Inhibition. Cancer Res..

[75] Kothapalli D., Zhao L., Hawthorne E.A., Cheng Y., Lee E., Pure E., Assoian R.K. (2007). Hyaluronan and CD44 antagonize mitogen-dependent cyclin D1 expression in mesenchymal cells. J. Cell Biol..

[76] Zhou W., Srinivasan S., Nawaz Z., Slingerland J.M. (2014). ER alpha, SKP2 and E2F-1 form a feed forward loop driving late ER alpha targets and G1 cell cycle progression. Oncogene.

[77] Bell E., Lunec J., Tweddle D.A. (2007). Cell cycle regulation targets of MYCN identified by gene expression microarrays. Cell Cycle.

[78] Cox B.D., Natarajan M., Stettner M.R., Gladson C.L. (2006). New concepts regarding focal adhesion kinase promotion of cell migration and proliferation. J. Cell. Biochem..

[79] Beierle E.A., Trujillo A., Nagaram A., Kurenova E.V., Finch R., Ma X., Vella J., Cance W.G., Golubovskaya V.M. (2007). N-MYC regulates focal adhesion kinase expression in human neuroblastoma. J. Biol. Chem..

[80] Stafman L.L., Williams A.P., Marayati R., Aye J.M., Markert H.R., Garner E.F., Quinn C.H., Lallani S.B., Stewart J.E., Yoon K.J. (2019). Focal Adhesion Kinase Inhibition Contributes to Tumor Cell Survival and Motility in Neuroblastoma Patient-Derived Xenografts. Sci. Rep..

[81] Mitra S.K., Schlaepfer D.D. (2006). Integrin-regulated FAK-Src signaling in normal and cancer cells. Curr. Opin. Cell Biol..

[82] Zhao J., Pestell R., Guan J.L. (2001). Transcriptional activation of cyclin D1 promoter by FAK contributes to cell cycle progression. Mol. Biol. Cell.

[83] Zhao J., Bian Z.C., Yee K., Chen B.P., Chien S., Guan J.L. (2003). Identification of transcription factor KLF8 as a downstream target of focal adhesion kinase in its regulation of cyclin D1 and cell cycle progression. Mol. Cell.

[84] Ding Q., Grammer J.R., Nelson M.A., Guan J.L., Stewart J.E., Gladson C.L. (2005). p27Kip1 and cyclin D1 are necessary for focal adhesion kinase regulation of cell cycle progression in glioblastoma cells propagated in vitro and in vivo in the scid mouse brain. J. Biol. Chem..

[85] Yu Y.T., Meier U.T. (2014). RNA-guided isomerization of uridine to pseudouridine—Pseudouridylation. RNA Biol..

[86] Zhang M., Pan Y., Jiang R., Hou P., Shan H., Chen F., Jiang T., Bai J., Zheng J. (2018). DKC1 serves as a potential prognostic biomarker for human clear cell renal cell carcinoma and promotes its proliferation, migration and invasion via the NF-κB pathway. Oncol. Rep..

[87] Miao F.A., Chu K., Chen H.R., Zhang M., Shi P.C., Bai J., You Y.P. (2019). Increased DKC1 expression in glioma and its significance in tumor cell proliferation, migration and invasion. Investig. New Drugs.

[88] O’Brien R., Tran S.L., Maritz M.F., Liu B., Kong C.F., Purgato S., Yang C., Murray J., Russell A.J., Flemming C.L. (2016). MYC-Driven Neuroblastomas Are Addicted to a Telomerase-Independent Function of Dyskerin. Cancer Res..

[89] Liu B., Zhang J., Huang C., Liu H. (2012). Dyskerin overexpression in human hepatocellular carcinoma is associated with advanced clinical stage and poor patient prognosis. PLoS ONE.

[90] Bellodi C., Krasnykh O., Haynes N., Theodoropoulou M., Peng G., Montanaro L., Ruggero D. (2010). Loss of function of the tumor suppressor DKC1 perturbs p27 translation control and contributes to pituitary tumorigenesis. Cancer Res..

[91] Yoon A., Peng G., Brandenburger Y., Zollo O., Xu W., Rego E., Ruggero D. (2006). Impaired control of IRES-mediated translation in X-linked dyskeratosis congenita. Science.

[92] He J., Gu L., Zhang H., Zhou M. (2011). Crosstalk between MYCN and MDM2-p53 signal pathways regulates tumor cell growth and apoptosis in neuroblastoma. Cell Cycle.

[93] Chen P.C., Yen C.C., Hung G.Y., Pan C.C., Chen W.M. (2019). Gene amplification and tumor grading in parosteal osteosarcoma. J. Chin. Med. Assoc..

[94] Deb S.P., Singh S., Deb S. (2014). MDM2 overexpression, activation of signaling networks, and cell proliferation. Subcell. Biochem..

[95] Slack A., Chen Z., Tonelli R., Pule M., Hunt L., Pession A., Shohet J.M. (2005). The p53 regulatory gene MDM2 is a direct transcriptional target of MYCN in neuroblastoma. Proc. Natl. Acad. Sci. USA.

[96] Zhu S., Lee J.S., Guo F., Shin J., Perez-Atayde A.R., Kutok J.L., Rodig S.J., Neuberg D.S., Helman D., Feng H. (2012). Activated ALK collaborates with MYCN in neuroblastoma pathogenesis. Cancer Cell.

[97] Brown D.R., Thomas C.A., Deb S.P. (1998). The human oncoprotein MDM2 arrests the cell cycle: Elimination of its cell-cycle-inhibitory function induces tumorigenesis. EMBO J..

[98] Deb S.P. (2003). Cell cycle regulatory functions of the human oncoprotein MDM2. Mol. Cancer Res..

[99] Chen J., Wu X., Lin J., Levine A.J. (1996). mdm-2 inhibits the G1 arrest and apoptosis functions of the p53 tumor suppressor protein. Mol. Cell. Biol..

[100] Marine J.C., Lozano G. (2010). Mdm2-mediated ubiquitylation: p53 and beyond. Cell Death Differ..

[101] Zhao Y., Yu H., Hu W. (2014). The regulation of MDM2 oncogene and its impact on human cancers. Acta Biochim. Biophys. Sin..

[102] Zhang Y., Xiong Y., Yarbrough W.G. (1998). ARF promotes MDM2 degradation and stabilizes p53: ARF-INK4a locus deletion impairs both the Rb and p53 tumor suppression pathways. Cell.

[103] Pomerantz J., Schreiber-Agus N., Liegeois N.J., Silverman A., Alland L., Chin L., Potes J., Chen K., Orlow I., Lee H.W. (1998). The Ink4a tumor suppressor gene product, p19Arf, interacts with MDM2 and neutralizes MDM2’s inhibition of p53. Cell.

[104] Stott F.J., Bates S., James M.C., McConnell B.B., Starborg M., Brookes S., Palmero I., Ryan K., Hara E., Vousden K.H. (1998). The alternative product from the human CDKN2A locus, p14(ARF), participates in a regulatory feedback loop with p53 and MDM2. EMBO J..

[105] Yap D.B., Hsieh J.K., Chan F.S., Lu X. (1999). mdm2: A bridge over the two tumour suppressors, p53 and Rb. Oncogene.

[106] Zhang Z., Wang H., Li M., Rayburn E.R., Agrawal S., Zhang R. (2005). Stabilization of E2F1 protein by MDM2 through the E2F1 ubiquitination pathway. Oncogene.

[107] Bell L.A., Ryan K.M. (2004). Life and death decisions by E2F-1. Cell Death Differ..

[108] Cao J., Jiang X., Peng X. (2018). Forkhead box M1 inhibits endothelial cell apoptosis and cell-cycle arrest through ROS generation. Int. J. Clin. Exp. Pathol..

[109] Laoukili J., Stahl M., Medema R.H. (2007). FoxM1: At the crossroads of ageing and cancer. Biochim. Biophys. Acta.

[110] Halasi M., Gartel A.L. (2013). FOX(M1) news—It is cancer. Mol. Cancer Ther..

[111] Vanhauwaert S., Decaesteker B., De Brouwer S., Leonelli C., Durinck K., Mestdagh P., Vandesompele J., Sermon K., Denecker G., Van Neste C. (2018). In silico discovery of a FOXM1 driven embryonal signaling pathway in therapy resistant neuroblastoma tumors. Sci. Rep..

[112] Kelleher F.C., O’Sullivan H. (2016). FOXM1 in sarcoma: Role in cell cycle, pluripotency genes and stem cell pathways. Oncotarget.

[113] Liao J., Jiang L., Wang C., Zhao D., He W., Zhou K., Liang Y. (2022). FoxM1 Regulates Proliferation and Apoptosis of Human Neuroblastoma Cell through PI3K/AKT Pathway. Fetal Pediatr. Pathol..

[114] Costa R.H. (2005). FoxM1 dances with mitosis. Nat. Cell Biol..

[115] Costa R.H., Kalinichenko V.V., Holterman A.X., Wang X. (2003). Transcription factors in liver development, differentiation, and regeneration. Hepatology.

[116] Leung T.W., Lin S.S., Tsang A.C., Tong C.S., Ching J.C., Leung W.Y., Gimlich R., Wong G.G., Yao K.M. (2001). Over-expression of FoxM1 stimulates cyclin B1 expression. FEBS Lett..

[117] Wang X., Quail E., Hung N.J., Tan Y., Ye H., Costa R.H. (2001). Increased levels of forkhead box M1B transcription factor in transgenic mouse hepatocytes prevent age-related proliferation defects in regenerating liver. Proc. Natl. Acad. Sci. USA.

[118] Lam E.W., Brosens J.J., Gomes A.R., Koo C.Y. (2013). Forkhead box proteins: Tuning forks for transcriptional harmony. Nat. Rev. Cancer.

[119] Fu Z., Malureanu L., Huang J., Wang W., Li H., van Deursen J.M., Tindall D.J., Chen J. (2008). Plk1-dependent phosphorylation of FoxM1 regulates a transcriptional programme required for mitotic progression. Nat. Cell Biol..

[120] Rizki A., Mott J.D., Bissell M.J. (2007). Polo-like kinase 1 is involved in invasion through extracellular matrix. Cancer Res..

[121] Bahassi E. (2011). Polo-like kinases and DNA damage checkpoint: Beyond the traditional mitotic functions. Exp. Biol. Med..

[122] Xiao D.B., Yue M., Su H.X., Ren P., Jiang J., Li F., Hu Y.F., Du H.N., Liu H.D., Qing G.L. (2016). Polo-like Kinase-1 Regulates Myc Stabilization and Activates a Feedforward Circuit Promoting Tumor Cell Survival. Mol. Cell.

[123] Schmucker S., Sumara I. (2014). Molecular dynamics of PLK1 during mitosis. Mol. Cell. Oncol..

[124] Kumar S., Sharma A.R., Sharma G., Chakraborty C., Kim J. (2016). PLK-1: Angel or devil for cell cycle progression. Biochim. Biophys. Acta.

[125] Casenghi M., Meraldi P., Weinhart U., Duncan P.I., Korner R., Nigg E.A. (2003). Polo-like kinase 1 regulates Nlp, a centrosome protein involved in microtubule nucleation. Dev. Cell.

[126] Lee K., Rhee K. (2011). PLK1 phosphorylation of pericentrin initiates centrosome maturation at the onset of mitosis. J. Cell Biol..

[127] Mardin B.R., Agircan F.G., Lange C., Schiebel E. (2011). Plk1 controls the Nek2A-PP1gamma antagonism in centrosome disjunction. Curr. Biol..

[128] Roshak A.K., Capper E.A., Imburgia C., Fornwald J., Scott G., Marshall L.A. (2000). The human polo-like kinase, PLK, regulates cdc2/cyclin B through phosphorylation and activation of the cdc25C phosphatase. Cell. Signal..

[129] Inoue D., Sagata N. (2005). The Polo-like kinase Plx1 interacts with and inhibits Myt1 after fertilization of *Xenopus* eggs. EMBO J..

[130] Watanabe N., Arai H., Nishihara Y., Taniguchi M., Watanabe N., Hunter T., Osada H. (2004). M-phase kinases induce phospho-dependent ubiquitination of somatic Wee1 by SCFbeta-TrCP. Proc. Natl. Acad. Sci. USA.

[131] Yuan J., Eckerdt F., Bereiter-Hahn J., Kurunci-Csacsko E., Kaufmann M., Strebhardt K. (2002). Cooperative phosphorylation including the activity of polo-like kinase 1 regulates the subcellular localization of cyclin B1. Oncogene.

[132] Sumara I., Vorlaufer E., Stukenberg P.T., Kelm O., Redemann N., Nigg E.A., Peters J.M. (2002). The dissociation of cohesin from chromosomes in prophase is regulated by polo-like kinase. Mol. Cell.

[133] Kitajima T.S., Sakuno T., Ishiguro K., Iemura S., Natsume T., Kawashima S.A., Watanabe Y. (2006). Shugoshin collaborates with protein phosphatase 2A to protect cohesin. Nature.

[134] Zhang Q., Liu H. (2020). Functioning mechanisms of Shugoshin-1 in centromeric cohesion during mitosis. Essays Biochem..

[135] Hansen D.V., Loktev A.V., Ban K.H., Jackson P.K. (2004). Plk1 regulates activation of the anaphase promoting complex by phosphorylating and triggering SCFbetaTrCP-dependent destruction of the APC Inhibitor Emi1. Mol. Biol. Cell.

[136] Moshe Y., Boulaire J., Pagano M., Hershko A. (2004). Role of Polo-like kinase in the degradation of early mitotic inhibitor 1, a regulator of the anaphase promoting complex/cyclosome. Proc. Natl. Acad. Sci. USA.

[137] Nasmyth K. (2002). Segregating sister genomes: The molecular biology of chromosome separation. Science.

[138] Hornig N.C., Uhlmann F. (2004). Preferential cleavage of chromatin-bound cohesin after targeted phosphorylation by Polo-like kinase. EMBO J..

[139] Kakeno M., Matsuzawa K., Matsui T., Akita H., Sugiyama I., Ishidate F., Nakano A., Takashima S., Goto H., Inagaki M. (2014). Plk1 Phosphorylates CLIP-170 and Regulates Its Binding to Microtubules for Chromosome Alignment. Cell Struct. Funct..

[140] Burkard M.E., Maciejowski J., Rodriguez-Bravo V., Repka M., Lowery D.M., Clauser K.R., Zhang C., Shokat K.M., Carr S.A., Yaffe M.B. (2009). Plk1 Self-Organization and Priming Phosphorylation of HsCYK-4 at the Spindle Midzone Regulate the Onset of Division in Human Cells. PLoS Biol..

[141] Neef R., Gruneberg U., Kopajtich R., Li X.L., Nigg E.A., Sillje H., Barr F.A. (2007). Choice of Plk1 docking partners during mitosis and cytokinesis is controlled by the activation state of Cdk1. Nat. Cell Biol..

[142] Hu C.K., Ozlu N., Coughlin M., Steen J.J., Mitchison T.J. (2012). Plk1 negatively regulates PRC1 to prevent premature midzone formation before cytokinesis. Mol. Biol. Cell.

[143] Wezensky S.J., Hanks T.S., Wilkison M.J., Ammons M.C., Siemsen D.W., Gauss K.A. (2010). Modulation of PLAGL2 transactivation by positive cofactor 2 (PC2), a component of the ARC/Mediator complex. Gene.

[144] Wang L., Sun L., Liu R., Mo H., Niu Y., Chen T., Wang Y., Han S., Tu K., Liu Q. (2021). Long non-coding RNA MAPKAPK5-AS1/PLAGL2/HIF-1α signaling loop promotes hepatocellular carcinoma progression. J. Exp. Clin. Cancer Res..

[145] Zheng H., Ying H., Wiedemeyer R., Yan H., Quayle S.N., Ivanova E.V., Paik J.H., Zhang H., Xiao Y., Perry S.R. (2010). PLAGL2 regulates Wnt signaling to impede differentiation in neural stem cells and gliomas. Cancer Cell.

[146] Li N., Li D., Du Y., Su C., Yang C., Lin C., Li X., Hu G. (2019). Overexpressed PLAGL2 transcriptionally activates Wnt6 and promotes cancer development in colorectal cancer. Oncol. Rep..

[147] Landrette S.F., Kuo Y.H., Hensen K., van Waalwijk van Doorn-Khosrovani S.B., Perrat P.N., van de Ven W.J., Delwel R., Castilla L.H. (2005). Plag1 and Plagl2 are oncogenes that induce acute myeloid leukemia in cooperation with Cbfb-MYH11. Blood.

[148] Zhao Z., Shelton S.D., Oviedo A., Baker A.L., Bryant C.P., Omidvarnia S., Du L. (2020). The PLAGL2/MYCN/miR-506-3p interplay regulates neuroblastoma cell fate and associates with neuroblastoma progression. J. Exp. Clin. Cancer Res..

[149] Hanks T.S., Gauss K.A. (2012). Pleomorphic adenoma gene-like 2 regulates expression of the p53 family member, p73, and induces cell cycle block and apoptosis in human promonocytic U937 cells. Apoptosis.

[150] Zheng G., Ning J., Yang Y.C. (2007). PLAGL2 controls the stability of Pirh2, an E3 ubiquitin ligase for p53. Biochem. Biophys. Res. Commun..

[151] Celeghin A., Giunco S., Freguja R., Zangrossi M., Nalio S., Dolcetti R., De Rossi A. (2016). Short-term inhibition of TERT induces telomere length-independent cell cycle arrest and apoptotic response in EBV-immortalized and transformed B cells. Cell Death Dis..

[152] Jagadeesh S., Banerjee P.P. (2006). Telomerase reverse transcriptase regulates the expression of a key cell cycle regulator, cyclin D1. Biochem. Biophys. Res. Commun..

[153] Murphy D.M., Buckley P.G., Bryan K., Watters K.M., Koster J., van Sluis P., Molenaar J., Versteeg R., Stallings R.L. (2011). Dissection of the oncogenic MYCN transcriptional network reveals a large set of clinically relevant cell cycle genes as drivers of neuroblastoma tumorigenesis. Mol. Carcinog..

[154] Li Y., Zhou W., Wei L., Jin J., Tang K., Li C., Teh B.T., Chen X. (2012). The effect of Aurora kinases on cell proliferation, cell cycle regulation and metastasis in renal cell carcinoma. Int. J. Oncol..

[155] Giet R., Glover D.M. (2001). Drosophila aurora B kinase is required for histone H3 phosphorylation and condensin recruitment during chromosome condensation and to organize the central spindle during cytokinesis. J. Cell Biol..

[156] Kallio M.J., McCleland M.L., Stukenberg P.T., Gorbsky G.J. (2002). Inhibition of aurora B kinase blocks chromosome segregation, overrides the spindle checkpoint, and perturbs microtubule dynamics in mitosis. Curr. Biol..

[157] Alptekin A., Ye B., Yu Y., Poole C.J., van Riggelen J., Zha Y., Ding H.F. (2019). Glycine decarboxylase is a transcriptional target of MYCN required for neuroblastoma cell proliferation and tumorigenicity. Oncogene.

[158] Yuan Y., Sun L., Wang X., Chen J., Jia M., Zou Y., Sa H., Cai Y., Xu Y., Sun C. (2019). Identification of a new GLDC gene alternative splicing variant and its protumorigenic roles in lung cancer. Future Oncol..

[159] Kume A.K.H., Sakakibara T., Ishiguro Y., Kure S., Hiraga K. (1991). The glycine cleavage system. Molecular cloning of the chicken and human glycine decarboxylase cDNAs and some characteristics involved in the deduced protein structures. J. Biol. Chem..

[160] Tibbetts A.S., Appling D.R. (2010). Compartmentalization of Mammalian folate-mediated one-carbon metabolism. Annu. Rev. Nutr..

[161] Murakami-Tonami Y., Ikeda H., Yamagishi R., Inayoshi M., Inagaki S., Kishida S., Komata Y., Jan K., Takeuchi I., Kondo Y. (2016). SGO1 is involved in the DNA damage response in MYCN-amplified neuroblastoma cells. Sci. Rep..

[162] Chen Q., Wan X., Chen Y., Liu C., Gu M., Wang Z. (2019). SGO1 induces proliferation and metastasis of prostate cancer through AKT-mediated signaling pathway. Am. J. Cancer Res..

[163] Matsuura S., Kahyo T., Shinmura K., Iwaizumi M., Yamada H., Funai K., Kobayashi J., Tanahashi M., Niwa H., Ogawa H. (2013). SGOL1 variant B induces abnormal mitosis and resistance to taxane in non-small cell lung cancers. Sci. Rep..

[164] Wang Y.Q., Wang X.Y., Han L.W., Hu D.D. (2020). LncRNA MALAT1 Regulates the Progression and Cisplatin Resistance of Ovarian Cancer Cells via Modulating miR-1271-5p/E2F5 Axis. Cancer Manag. Res..

[165] Xu X., Cai N., Zhi T., Bao Z., Wang D., Liu Y., Jiang K., Fan L., Ji J., Liu N. (2017). MicroRNA-1179 inhibits glioblastoma cell proliferation and cell cycle progression via directly targeting E2F transcription factor 5. Am. J. Cancer Res..

[166] Hijmans E.M., Voorhoeve P.M., Beijersbergen R.L., van’t Veer L.J., Bernards R. (1995). E2F-5, a new E2F family member that interacts with p130 in vivo. Mol. Cell. Biol..

[167] Chen Q., Liang D., Overbeek P.A. (2008). Overexpression of E2F5/p130, but not E2F5 alone, can inhibit E2F-induced cell cycle entry in transgenic mice. Mol. Vis..

[168] Chen D., Cox J., Annam J., Weingart M., Essien G., Rathi K.S., Rokita J.L., Khurana P., Cuya S.M., Bosse K.R. (2020). LIN28B promotes neuroblastoma metastasis and regulates PDZ binding kinase. Neoplasia.

[169] Hu F., Gartenhaus R.B., Eichberg D., Liu Z., Fang H.B., Rapoport A.P. (2010). PBK/TOPK interacts with the DBD domain of tumor suppressor p53 and modulates expression of transcriptional targets including p21. Oncogene.

[170] Brooks W.S., Banerjee S., Crawford D.F. (2007). G2E3 is a nucleo-cytoplasmic shuttling protein with DNA damage responsive localization. Exp. Cell Res..

[171] Abe Y., Takeuchi T., Kagawa-Miki L., Ueda N., Shigemoto K., Yasukawa M., Kito K. (2007). A mitotic kinase TOPK enhances Cdk1/cyclin B1-dependent phosphorylation of PRC1 and promotes cytokinesis. J. Mol. Biol..

[172] El-Deiry W.S., Tokino T., Velculescu V.E., Levy D.B., Parsons R., Trent J.M., Lin D., Mercer W.E., Kinzler K.W., Vogelstein B. (1993). WAF1, a potential mediator of p53 tumor suppression. Cell.

[173] Rajbhandari P., Lopez G., Capdevila C., Salvatori B., Yu J., Rodriguez-Barrueco R., Martinez D., Yarmarkovich M., Weichert-Leahey N., Abraham B.J. (2018). Cross-Cohort Analysis Identifies a TEAD4-MYCN Positive Feedback Loop as the Core Regulatory Element of High-Risk Neuroblastoma. Cancer Discov..

[174] Zhang W.C., Shyh-Chang N., Yang H., Rai A., Umashankar S., Ma S., Soh B.S., Sun L.L., Tai B.C., Nga M.E. (2012). Glycine decarboxylase activity drives non-small cell lung cancer tumor-initiating cells and tumorigenesis. Cell.

[175] Min H.L., Kim J., Kim W.H., Jang B.G., Kim M.A. (2016). Epigenetic Silencing of the Putative Tumor Suppressor Gene GLDC (Glycine Dehydrogenase) in Gastric Carcinoma. Anticancer Res..

[176] Haugen B.R., Nawaz S., Markham N., Hashizumi T., Shroyer A.L., Werness B., Shroyer K.R. (1997). Telomerase activity in benign and malignant thyroid tumors. Thyroid.

[177] Liang W., Ye D., Dai L., Shen Y., Xu J. (2012). Overexpression of hTERT extends replicative capacity of human nucleus pulposus cells, and protects against serum starvation-induced apoptosis and cell cycle arrest. J. Cell. Biochem..

[178] Martínez P., Blasco M.A. (2011). Telomeric and extra-telomeric roles for telomerase and the telomere-binding proteins. Nat. Rev. Cancer.

[179] Nandi A., Tidwell M., Karp J., Rapoport A.P. (2004). Protein expression of PDZ-binding kinase is up-regulated in hematologic malignancies and strongly down-regulated during terminal differentiation of HL-60 leukemic cells. Blood Cells Mol. Dis..

[180] Côté S., Simard C., Lemieux R. (2002). Regulation of growth-related genes by interleukin-6 in murine myeloma cells. Cytokine.

[181] Simons-Evelyn M., Bailey-Dell K., Toretsky J.A., Ross D.D., Fenton R., Kalvakolanu D., Rapoport A.P. (2001). PBK/TOPK is a novel mitotic kinase which is upregulated in Burkitt’s lymphoma and other highly proliferative malignant cells. Blood Cells Mol. Dis..

[182] Li M., Zhou J.Y., Ge Y., Matherly L.H., Wu G.S. (2003). The phosphatase MKP1 is a transcriptional target of p53 involved in cell cycle regulation. J. Biol. Chem..

[183] Lee J., Kitajima T.S., Tanno Y., Yoshida K., Morita T., Miyano T., Miyake M., Watanabe Y. (2008). Unified mode of centromeric protection by shugoshin in mammalian oocytes and somatic cells. Nat. Cell Biol..

[184] Liu H., Jia L., Yu H. (2013). Phospho-H2A and cohesin specify distinct tension-regulated Sgo1 pools at kinetochores and inner centromeres. Curr. Biol..

[185] Liu H., Qu Q., Warrington R., Rice A., Cheng N., Yu H. (2015). Mitotic Transcription Installs Sgo1 at Centromeres to Coordinate Chromosome Segregation. Mol. Cell.

[186] Liu L., Zhang N., Liu J., Min J., Ma N., Liu N., Liu Y., Zhang H. (2012). Lentivirus-mediated siRNA interference targeting SGO-1 inhibits human NSCLC cell growth. Tumour Biol..

[187] Yamada H.Y., Yao Y., Wang X., Zhang Y., Huang Y., Dai W., Rao C.V. (2012). Haploinsufficiency of SGO1 results in deregulated centrosome dynamics, enhanced chromosomal instability and colon tumorigenesis. Cell Cycle.

[188] Yang J., Ikezoe T., Nishioka C., Yokoyama A. (2013). A novel treatment strategy targeting shugoshin 1 in hematological malignancies. Leuk. Res..

[189] Vader G., Medema R.H., Lens S.M. (2006). The chromosomal passenger complex: Guiding Aurora-B through mitosis. J. Cell Biol..

[190] Chieffi P. (2018). Aurora B: A new promising therapeutic target in cancer. Intractable Rare Dis. Res..

[191] Bogen D., Wei J.S., Azorsa D.O., Ormanoglu P., Buehler E., Guha R., Keller J.M., Mathews Griner L.A., Ferrer M., Song Y.K. (2015). Aurora B kinase is a potent and selective target in MYCN-driven neuroblastoma. Oncotarget.

[192] Terada Y., Tatsuka M., Suzuki F., Yasuda Y., Fujita S., Otsu M. (1998). AIM-1: A mammalian midbody-associated protein required for cytokinesis. EMBO J..

[193] Xie H., Kang Y., Wang S., Zheng P., Chen Z., Roy S., Zhao C. (2020). E2f5 is a versatile transcriptional activator required for spermatogenesis and multiciliated cell differentiation in zebrafish. PLoS Genet..

[194] Liu Y., Liu D.H., Wan W.Q. (2019). MYCN-induced E2F5 promotes neuroblastoma cell proliferation through regulating cell cycle progression. Biochem. Bioph. Res. Commun..

[195] Chen M., Huang B., Zhu L., Chen K., Liu M., Zhong C. (2020). Structural and Functional Overview of TEAD4 in Cancer Biology. OncoTargets Ther..

[196] Liu X., Li H., Rajurkar M., Li Q., Cotton J.L., Ou J., Zhu L.J., Goel H.L., Mercurio A.M., Park J.S. (2016). Tead and AP1 Coordinate Transcription and Motility. Cell Rep..

[197] Park J.A., Cheung N.V. (2020). Targets and Antibody Formats for Immunotherapy of Neuroblastoma. J. Clin. Oncol..

[198] Whittle S.B., Smith V., Doherty E., Zhao S., McCarty S., Zage P.E. (2017). Overview and recent advances in the treatment of neuroblastoma. Expert Rev. Anticancer Ther..

[199] Ara T., DeClerck Y.A. (2006). Mechanisms of invasion and metastasis in human neuroblastoma. Cancer Metastasis Rev..

[200] Maris J.M. (2010). Recent advances in neuroblastoma. N. Engl. J. Med..

[201] Mlakar V., Jurkovic Mlakar S., Lopez G., Maris J.M., Ansari M., Gumy-Pause F. (2017). 11q deletion in neuroblastoma: A review of biological and clinical implications. Mol. Cancer.

[202] Yue Z.X., Huang C., Gao C., Xing T.Y., Liu S.G., Li X.J., Zhao Q., Wang X.S., Zhao W., Jin M. (2017). MYCN amplification predicts poor prognosis based on interphase fluorescence in situ hybridization analysis of bone marrow cells in bone marrow metastases of neuroblastoma. Cancer Cell Int..

[203] Gonzalez Curto G., der Vartanian A., Frarma Y.E., Manceau L., Baldi L., Prisco S., Elarouci N., Causeret F., Korenkov D., Rigolet M. (2020). The PAX-FOXO1s trigger fast trans-differentiation of chick embryonic neural cells into alveolar rhabdomyosarcoma with tissue invasive properties limited by S phase entry inhibition. PLoS Genet..

[204] Li J., Kretzner L. (2003). The growth-inhibitory Ndrg1 gene is a Myc negative target in human neuroblastomas and other cell types with overexpressed N- or c-myc. Mol. Cell. Biochem..

[205] Akiba J., Murakami Y., Noda M., Watari K., Ogasawara S., Yoshida T., Kawahara A., Sanada S., Yasumoto M., Yamaguchi R. (2011). N-myc downstream regulated gene1/Cap43 overexpression suppresses tumor growth by hepatic cancer cells through cell cycle arrest at the G0/G1 phase. Cancer Lett..

[206] Kovacevic Z., Sivagurunathan S., Mangs H., Chikhani S., Zhang D., Richardson D.R. (2011). The metastasis suppressor, N-myc downstream regulated gene 1 (NDRG1), upregulates p21 via p53-independent mechanisms. Carcinogenesis.

[207] Chen Z., Zhang D., Yue F., Zheng M., Kovacevic Z., Richardson D.R. (2012). The iron chelators Dp44mT and DFO inhibit TGF-beta-induced epithelial-mesenchymal transition via up-regulation of N-Myc downstream-regulated gene 1 (NDRG1). J. Biol. Chem..

[208] Fotovati A., Fujii T., Yamaguchi M., Kage M., Shirouzu K., Oie S., Basaki Y., Ono M., Yamana H., Kuwano M. (2006). 17Beta-estradiol induces down-regulation of Cap43/NDRG1/Drg-1, a putative differentiation-related and metastasis suppressor gene, in human breast cancer cells. Clin. Cancer Res..

[209] Petroni M., Sardina F., Heil C., Sahún-Roncero M., Colicchia V., Veschi V., Albini S., Fruci D., Ricci B., Soriani A. (2016). The MRN complex is transcriptionally regulated by MYCN during neural cell proliferation to control replication stress. Cell Death Differ..

[210] Stracker T.H., Petrini J.H. (2011). The MRE11 complex: Starting from the ends. Nat. Rev. Mol. Cell Biol..

[211] Jin M.H., Oh D.Y. (2019). ATM in DNA repair in cancer. Pharmacol. Ther..

[212] Puissant A., Frumm S.M., Alexe G., Bassil C.F., Qi J., Chanthery Y.H., Nekritz E.A., Zeid R., Gustafson W.C., Greninger P. (2013). Targeting MYCN in neuroblastoma by BET bromodomain inhibition. Cancer Discov..

[213] Chipumuro E., Marco E., Christensen C.L., Kwiatkowski N., Zhang T., Hatheway C.M., Abraham B.J., Sharma B., Yeung C., Altabef A. (2014). CDK7 inhibition suppresses super-enhancer-linked oncogenic transcription in MYCN-driven cancer. Cell.

[214] Poon E., Liang T., Jamin Y., Walz S., Kwok C., Hakkert A., Barker K., Urban Z., Thway K., Zeid R. (2020). Orally bioavailable CDK9/2 inhibitor shows mechanism-based therapeutic potential in MYCN-driven neuroblastoma. J. Clin. Investig..

[215] Chesler L., Schlieve C., Goldenberg D.D., Kenney A., Kim G., McMillan A., Matthay K.K., Rowitch D., Weiss W.A. (2006). Inhibition of phosphatidylinositol 3-kinase destabilizes Mycn protein and blocks malignant progression in neuroblastoma. Cancer Res..

[216] Ackermann S., Goeser F., Schulte J.H., Schramm A., Ehemann V., Hero B., Eggert A., Berthold F., Fischer M. (2011). Polo-like kinase 1 is a therapeutic target in high-risk neuroblastoma. Clin. Cancer Res..

[217] Park J.H., Szemes M., Vieira G.C., Melegh Z., Malik S., Heesom K.J., Von Wallwitz-Freitas L., Greenhough A., Brown K.W., Zheng Y.G. (2015). Protein arginine methyltransferase 5 is a key regulator of the MYCN oncoprotein in neuroblastoma cells. Mol. Oncol..

[218] Liu X., He J.Z., Mao L.B., Zhang Y.Y., Cui W.W., Duan S.J., Jiang A.L., Gao Y., Sang Y., Huang G.F. (2021). EPZ015666, a selective protein arginine methyltransferase 5 (PRMT5) inhibitor with an antitumour effect in retinoblastoma. Exp. Eye Res..

[219] Müller I., Larsson K., Frenzel A., Oliynyk G., Zirath H., Prochownik E.V., Westwood N.J., Henriksson M.A. (2014). Targeting of the MYCN protein with small molecule c-MYC inhibitors. PLoS ONE.

[220] Zirath H., Frenzel A., Oliynyk G., Segerström L., Westermark U.K., Larsson K., Munksgaard Persson M., Hultenby K., Lehtiö J., Einvik C. (2013). MYC inhibition induces metabolic changes leading to accumulation of lipid droplets in tumor cells. Proc. Natl. Acad. Sci. USA.

[221] Han H.Y., Jain A.D., Truica M.I., Izquierdo-Ferrer J., Anker J.F., Lysy B., Sagar V., Luan Y., Chalmers Z.R., Unno K. (2019). Small-Molecule MYC Inhibitors Suppress Tumor Growth and Enhance Immunotherapy. Cancer Cell.

[222] Struntz N.B., Chen A., Deutzmann A., Wilson R.M., Stefan E., Evans H.L., Ramirez M.A., Liang T., Caballero F., Wildschut M.H.E. (2019). Stabilization of the Max Homodimer with a Small Molecule Attenuates Myc-Driven Transcription. Cell Chem. Biol..

[223] Van Maerken T., Speleman F., Vermeulen J., Lambertz I., de Clercq S., de Smet E., Yigit N., Coppens V., Philippé J., de Paepe A. (2006). Small-molecule MDM2 antagonists as a new therapy concept for neuroblastoma. Cancer Res..

[224] Filippakopoulos P., Qi J., Picaud S., Shen Y., Smith W.B., Fedorov O., Morse E.M., Keates T., Hickman T.T., Felletar I. (2010). Selective inhibition of BET bromodomains. Nature.

[225] Kwiatkowski N., Zhang T., Rahl P.B., Abraham B.J., Reddy J., Ficarro S.B., Dastur A., Amzallag A., Ramaswamy S., Tesar B. (2014). Targeting transcription regulation in cancer with a covalent CDK7 inhibitor. Nature.

[226] Sánchez-Martínez C., Gelbert L.M., Lallena M.J., de Dios A. (2015). Cyclin dependent kinase (CDK) inhibitors as anticancer drugs. Bioorg. Med. Chem. Lett..

[227] Frame S., Saladino C., MacKay C., Atrash B., Sheldrake P., McDonald E., Clarke P.A., Workman P., Blake D., Zheleva D. (2020). Fadraciclib (CYC065), a novel CDK inhibitor, targets key pro-survival and oncogenic pathways in cancer. PLoS ONE.

[228] Kawakami M., Mustachio L.M., Rodriguez-Canales J., Mino B., Roszik J., Tong P., Wang J., Lee J.J., Myung J.H., Heymach J.V. (2017). Next-Generation CDK2/9 Inhibitors and Anaphase Catastrophe in Lung Cancer. J. Natl. Cancer Inst..

[229] Diolaiti D., McFerrin L., Carroll P.A., Eisenman R.N. (2015). Functional interactions among members of the MAX and MLX transcriptional network during oncogenesis. Biochim. Biophys. Acta.

[230] Braun C.J., Stanciu M., Boutz P.L., Patterson J.C., Calligaris D., Higuchi F., Neupane R., Fenoglio S., Cahill D.P., Wakimoto H. (2017). Coordinated Splicing of Regulatory Detained Introns within Oncogenic Transcripts Creates an Exploitable Vulnerability in Malignant Glioma. Cancer Cell.

[231] Chan-Penebre E., Kuplast K.G., Majer C.R., Boriack-Sjodin P.A., Wigle T.J., Johnston L.D., Rioux N., Munchhof M.J., Jin L., Jacques S.L. (2015). A selective inhibitor of PRMT5 with in vivo and in vitro potency in MCL models. Nat. Chem. Biol..

[232] Steegmaier M., Hoffmann M., Baum A., Lenart P., Petronczki M., Krssak M., Gurtler U., Garin-Chesa P., Lieb S., Quant J. (2007). BI 2536, a potent and selective inhibitor of polo-like kinase 1, inhibits tumor growth in vivo. Curr. Biol..

[233] Oliveira J.C., Pezuk J.A., Brassesco M.S., Morales A.G., Queiroz R.G., Scrideli C.A., Tone L.G. (2014). PLK1 expression and BI 2536 effects in childhood acute lymphoblastic leukemia. Pediatr. Blood Cancer.

[234] Murray M.J., Nicholson J.C., Coleman N. (2015). Biology of childhood germ cell tumours, focussing on the significance of microRNAs. Andrology.

[235] Vassilev L.T., Vu B.T., Graves B., Carvajal D., Podlaski F., Filipovic Z., Kong N., Kammlott U., Lukacs C., Klein C. (2004). In vivo activation of the p53 pathway by small-molecule antagonists of MDM2. Science.

[236] Yu Z., Zhuang C., Wu Y., Guo Z., Li J., Dong G., Yao J., Sheng C., Miao Z., Zhang W. (2014). Design, synthesis and biological evaluation of sulfamide and triazole benzodiazepines as novel p53-MDM2 inhibitors. Int. J. Mol. Sci..

[237] Daniele S., la Pietra V., Barresi E., di Maro S., da Pozzo E., Robello M., la Motta C., Cosconati S., Taliani S., Marinelli L. (2016). Lead Optimization of 2-Phenylindolylglyoxylyldipeptide Murine Double Minute (MDM)2/Translocator Protein (TSPO) Dual Inhibitors for the Treatment of Gliomas. J. Med. Chem..

