# Supplementary material for: A Review of the Regulatory Mechanisms of N-Myc on Cell Cycle
Source: Molecules. 2023 Jan 23;28(3):1141. doi: 10.3390/molecules28031141 (PMC9920120; doi:10.3390/molecules28031141)
Supplement: Supplementary file 1 [file molecules-28-01141-s001.zip › molecules-2010679-supplementary.pdf]

A review of the regulatory mechanisms of N-Myc on cell cycle

Hong-Li Li [1, 3], Lu-Lu Dong [2], Min-Jie Jin [2], Qian-Yu Li [2], Xiao Wang [2], Mei-Qi Jia [1], Jian Song [2,4,*] and Sai-Yang Zhang [2,*] and Shuo Yuan [1, 4,*]

1. Children's Hospital Affiliated of Zhengzhou University, Henan Children's Hospital, Zhengzhou Children's Hospital, Zhengzhou 450018, China
2. China School of Basic Medical Sciences, Zhengzhou University, Zhengzhou, 450001, China
3. Faculty of Laboratory Medicine, Zhengzhou University, Zhengzhou 450001, China
4. School of Pharmaceutical Sciences, Institute of Drug Discovery & Development, Zhengzhou University, Zhengzhou, 450001, China

* Correspondence: mumuandzz@163.com (J.S.); saiyangz@zzu.edu.cn (S.-Y.Z.); zzuyuanshuo@163.com (S.Y.)

Table S1. The summarization of 10 target genes of N-myc in section 2. These target genes regulated by N-Myc and further affecting the different process of cell cycle in multiple ways.

Target gene	Phase	Regulating target
POU5F1	G1/S<br>G2/M	Pou5f1 up-regulates the expression of *CCND* directly[1-3]. Pou5f1 can suppress the activities of CDK2 and cyclin D1/D2, and inhibit the phosphorylation of retinoblastoma protein RB by up-regulating the expression of miR-302[4, 5]. Pou5f1 up-regulates CDK4/6-Cyclin D to promote the hypo-phosphorylation of RB which restores the transcriptional activity of E2F to up-regulate *CCNE1/2* expression[6].Pou5f1 inhibit CDK1 activation by inhibiting the Cdc25[7]. Pou5f1 can maintain the stability of p53[7] and inhibit the activity of *p21,* a downstream target of p53, by directly binding to the *p21* promoter region or indirectly up-regulating *DNMT1[8].* Pou5f1 up-regulates CD49f[9].
PRMT1	G1	PRMT1 methylates C/EBPα, reducing the inhibitory effect on Cyclin D1[10]. PRMT1 can also regulate the expression of *CCNA2*, *CCNB1*, *CCND1*, *CCNE2*, *CDK6*, *CDC20*, and *CDC23*[10].
VRK1	G1/S	Knockdown of *VRK1* induces the down-regulation of Cyclin D1 or MDM2, leading to an increase in p53 and its target p21[11]. VRK1 can up-regulate CCND1 by phosphorylating histone H2A T120[12] and CREB[13]. The knockdown of *VRK1* also leads to the down-regulation of the BANF1, affecting mitotic nuclear reorganization[14, 15]. VRK1 up-regulates the expression of *CDK2* and *BIRC5*[16].
Skp2	G1/S	SKP2 triggers the degradation of p27, leading to the activation of Cyclin E-CDK2, meanwhile, the activated CDK2 causes the phosphorylation of RB, leading to the increased expression of SKP2[17-19]. SKP2 also targets cell cycle inhibitors (such as p27Kip1, p21Cip1, etc.) for ubiquitination and degradation[20-22].
PTK2	G1<br>G1/S	PTK2 up-regulated the transcription of *CCND1* directly or through the activation of the ERK pathway[23], and *CCND1* can also be directly activated by KLF8[24], which is regulated by PTK2. PTK2 can also reduce the expression of *CDKN1B* (p27Kip1) and *p21Waf1*, and enhance the activity of CDK4[25].
DKC1	G1	Dyskerin negatively regulates the expression of *CDK2* and *CCNE2*[26]. The impaired function of Dyskerin can affect the translation of specific mRNAs, including tumor suppressor p27[27]. The genetic interaction between *DKC1* and *p27* is required for cell cycle progression[28].
MDM2	G0/G1	Mdm2 competes with E3 ubiquitin ligase SCF^{Skp2} to bind to E2F1 and inhibits the ubiquitination of E2F1. In the early stage of G0/G1, When RB is phosphorylated, it can dissociate from E2F1, enabling cells in the late G1 phase to initiate cell cycle progression[29, 30]
FOXM1	G1/S<br>G2/M	FoxM1 directly or indirectly regulated the activation of target genes, such as *CCND*, *CDK4,* and *CCNE*, *CDK2* that induce cells to enter the S phase, Cyclin A-CDK1 involving in G2/M transition, and Cyclin B-CDK1 that induces the entry of M phase and promotes the process of mitosis[31-34]. In the G2/M phase, PLK1 combines with FoxM1 and directly phosphorylates it, thereby activating it to promote the expression of downstream mitotic regulators[35].
PLK1	G2/M<br>M	During the G2/M transition, Cdc25C is phosphorylated and activated by PLK1 and then activates CDK1 by dephosphorylating it, promoting the formation of Cyclin B1-CDK1 complex and ensuring mitotic entry[36]. PLK1 promotes sister chromatid segregation by activating the SA2 subunit of the binding protein and directly phosphorylating and activating APC/C[37-39]
PLAGL2	G1<br>G2/M	PLAGL2 affects its downstream cell cycle inhibitors p21 and p57 by regulating the expression level of TP73, inducing G1 phase arrest and a small amount of G2/M phase arrest[40]. PLAGL2 may regulate p53 by interacting with Pirh2, and indirectly participate in the regulation of cell cycle[41].

1. Su, C., *Survivin in survival of hepatocellular carcinoma.* Cancer Lett, 2016. **379**(2): p. 184-90.

102

2. Bai, M., M. Yuan, H. Liao, J. Chen, B. Xie, D. Yan, X. Xi, X. Xu, Z. Zhang, and Y. Feng, *OCT4 pseudogene 5 upregulates OCT4 expression to promote proliferation by competing with miR-145 in endometrial carcinoma.* Oncol Rep, 2015. **33**(4): p. 1745-52.

3. Han, S.M., S.H. Han, Y.R. Coh, G. Jang, J. Chan Ra, S.K. Kang, H.W. Lee, and H.Y. Youn, *Enhanced proliferation and differentiation of Oct4- and Sox2-overexpressing human adipose tissue mesenchymal stem cells.* Exp Mol Med, 2014. **46**(6): p. e101.

4. Card, D.A., P.B. Hebbar, L. Li, K.W. Trotter, Y. Komatsu, Y. Mishina, and T.K. Archer, *Oct4/Sox2-regulated miR-302 targets cyclin D1 in human embryonic stem cells.* Mol Cell Biol, 2008. **28**(20): p. 6426-38.

5. Lin, S.L., D.C. Chang, S.Y. Ying, D. Leu, and D.T. Wu, *MicroRNA miR-302 inhibits the tumorigenecity of human pluripotent stem cells by coordinate suppression of the CDK2 and CDK4/6 cell cycle pathways.* Cancer Res, 2010. **70**(22): p. 9473-82.

6. Pennycook, B.R. and A.R. Barr, *Restriction point regulation at the crossroads between quiescence and cell proliferation.* Febs Letters, 2020. **594**(13): p. 2046-2060.

7. She, S., Q. Wei, B. Kang, and Y.J. Wang, *Cell cycle and pluripotency: Convergence on octamer‑binding transcription factor 4 (Review).* Mol Med Rep, 2017. **16**(5): p. 6459-6466.

8. Bedford, M.T. and S.G. Clarke, *Protein arginine methylation in mammals: who, what, and why.* Mol Cell, 2009. **33**(1): p. 1-13.

9. Yu, K.R., S.R. Yang, J.W. Jung, H. Kim, K. Ko, D.W. Han, S.B. Park, S.W. Choi, S.K. Kang, H. Scholer, and K.S. Kang, *CD49f enhances multipotency and maintains stemness through the direct regulation of OCT4 and SOX2.* Stem Cells, 2012. **30**(5): p. 876-87.

10. Liu, L.M., W.Z. Sun, X.Z. Fan, Y.L. Xu, M.B. Cheng, and Y. Zhang, *Methylation of C/EBPα by PRMT1 Inhibits Its Tumor-Suppressive Function in Breast Cancer.* Cancer Res, 2019. **79**(11): p. 2865-2877.

11. Colmenero-Repiso, A., M.A. Gómez-Muñoz, I. Rodríguez-Prieto, A. Amador-Álvarez, K.O. Henrich, D. Pascual-Vaca, K. Okonechnikov, E. Rivas, F. Westermann, R. Pardal, and F.M. Vega, *Identification of VRK1 as a New Neuroblastoma Tumor Progression Marker Regulating Cell Proliferation.* Cancers (Basel), 2020. **12**(11).

12. Aihara, H., T. Nakagawa, H. Mizusaki, M. Yoneda, M. Kato, M. Doiguchi, Y. Imamura, M. Higashi, T. Ikura, T. Hayashi, Y. Kodama, M. Oki, T. Nakayama, E. Cheung, H. Aburatani, K.I. Takayama, H. Koseki, S. Inoue, Y. Takeshima, and T. Ito, *Histone H2A T120 Phosphorylation Promotes Oncogenic Transformation via Upregulation of Cyclin D1.* Mol Cell, 2016. **64**(1): p. 176-188.

13. Kang, T.H., D.Y. Park, W. Kim, and K.T. Kim, *VRK1 phosphorylates CREB and mediates CCND1 expression.* Journal of Cell Science, 2008. **121**(18): p. 3035-3041.

14. Ren, Z., J. Geng, C. Xiong, X. Li, Y. Li, J. Li, and H. Liu, *Downregulation of VRK1 reduces the expression of BANF1 and suppresses the proliferative and migratory activity of esophageal cancer cells.* Oncol Lett, 2020. **20**(2): p. 1163-1170.

15. Jamin, A., A. Wicklund, and M.S. Wiebe, *Cell- and virus-mediated regulation of the barrier-to-autointegration factor's phosphorylation state controls its DNA binding, dimerization, subcellular localization, and antipoxviral activity.* J Virol, 2014. **88**(10): p. 5342-55.

16. Santos, C.R., M. Rodríguez-Pinilla, F.M. Vega, J.L. Rodríguez-Peralto, S. Blanco, A. Sevilla, A. Valbuena, T. Hernández, A.J. van Wijnen, F. Li, E. de Alava, M. Sánchez-Céspedes, and P.A. Lazo, *VRK1 signaling pathway in the context of the proliferation phenotype in head and neck squamous cell carcinoma.* Mol Cancer Res, 2006. **4**(3): p. 177-85.

17. Assoian, R.K. and Y. Yung, *A reciprocal relationship between Rb and Skp2 - Implications for restriction point control, signal transduction to the cell cycle and cancer.* Cell Cycle, 2008. **7**(1): p. 24-27.

18. Hydbring, P., A. Castell, and L.G. Larsson, *MYC Modulation around the CDK2/p27/SKP2 Axis.* Genes, 2017. **8**(7).

19. Yung, Y., J.L. Walker, J.M. Roberts, and R.K. Assoian, *A Skp2 autoinduction loop and restriction point control.* Journal of Cell Biology, 2007. **178**(5): p. 741-747.

20. Deng, T., G. Yan, X. Song, L. Xie, Y. Zhou, J. Li, X. Hu, Z. Li, J. Hu, Y. Zhang, H. Zhang, Y. Sun, P. Feng, D. Wei, B. Hu, J. Liu, W. Tan, and M. Ye, *Deubiquitylation and stabilization of p21 by USP11 is critical for cell-cycle progression and DNA damage responses.* Proc Natl Acad Sci U S A, 2018. **115**(18): p. 4678-4683.

21. Jia, T., L. Zhang, Y. Duan, M. Zhang, G. Wang, J. Zhang, and Z. Zhao, *The differential susceptibilities of MCF-7 and MDA-MB-231 cells to the cytotoxic effects of curcumin are associated with the PI3K/Akt-SKP2-Cip/Kips pathway.* Cancer Cell Int, 2014. **14**(1): p. 126.

22. Bell, E., J. Lunec, and D.A. Tweddle, *Cell cycle regulation targets of MYCN identified by gene expression microarrays.* Cell Cycle, 2007. **6**(10): p. 1249-56.

23. Zhao, J., R. Pestell, and J.L. Guan, *Transcriptional activation of cyclin D1 promoter by FAK contributes to cell cycle progression.* Mol Biol Cell, 2001. **12**(12): p. 4066-77.

24. Zhao, J., Z.C. Bian, K. Yee, B.P. Chen, S. Chien, and J.L. Guan, *Identification of transcription factor KLF8 as a downstream target of focal adhesion kinase in its regulation of cyclin D1 and cell cycle progression.* Mol Cell, 2003. **11**(6): p. 1503-15.

25. Ding, Q., J.R. Grammer, M.A. Nelson, J.L. Guan, J.E. Stewart, Jr., and C.L. Gladson, *p27Kip1 and cyclin D1 are necessary for focal adhesion kinase regulation of cell cycle progression in glioblastoma cells propagated in vitro and in vivo in the scid mouse brain.* J Biol Chem, 2005. **280**(8): p. 6802-15.

26. Miao, F.A., K. Chu, H.R. Chen, M. Zhang, P.C. Shi, J. Bai, and Y.P. You, *Increased DKC1 expression in glioma and its significance in tumor cell proliferation, migration and invasion.* Invest New Drugs, 2019. **37**(6): p. 1177-1186.

27. Bellodi, C., O. Krasnykh, N. Haynes, M. Theodoropoulou, G. Peng, L. Montanaro, and D. Ruggero, *Loss of function of the tumor suppressor DKC1 perturbs p27 translation control and contributes to pituitary tumorigenesis.* Cancer Res, 2010. **70**(14): p. 6026-35.

28. Yoon, A., G. Peng, Y. Brandenburger, O. Zollo, W. Xu, E. Rego, and D. Ruggero, *Impaired control of IRES-mediated translation in X-linked dyskeratosis congenita.* Science, 2006. **312**(5775): p. 902-6.

29. Zhang, Z., H. Wang, M. Li, E.R. Rayburn, S. Agrawal, and R. Zhang, *Stabilization of E2F1 protein by MDM2 through the E2F1 ubiquitination pathway.* Oncogene, 2005. **24**(48): p. 7238-47.

007

30. Bell, L.A. and K.M. Ryan, *Life and death decisions by E2F-1.* Cell Death Differ, 2004. **11**(2): p. 137-42.

016

31. Laoukili, J., M. Stahl, and R.H. Medema, *FoxM1: at the crossroads of ageing and cancer.* Biochim Biophys Acta, 2007. **1775**(1): p. 92-102.

32. Costa, R.H., *FoxM1 dances with mitosis.* Nat Cell Biol, 2005. **7**(2): p. 108-10.

33. Costa, R.H., V.V. Kalinichenko, A.X. Holterman, and X. Wang, *Transcription factors in liver development, differentiation, and regeneration.* Hepatology, 2003. **38**(6): p. 1331-47.

34. Leung, T.W., S.S. Lin, A.C. Tsang, C.S. Tong, J.C. Ching, W.Y. Leung, R. Gimlich, G.G. Wong, and K.M. Yao, *Over-expression of FoxM1 stimulates cyclin B1 expression.* FEBS Lett, 2001. **507**(1): p. 59-66.

35. Fu, Z., L. Malureanu, J. Huang, W. Wang, H. Li, J.M. van Deursen, D.J. Tindall, and J. Chen, *Plk1-dependent phosphorylation of FoxM1 regulates a transcriptional programme required for mitotic progression.* Nat Cell Biol, 2008. **10**(9): p. 1076-82.

36. Roshak, A.K., E.A. Capper, C. Imburgia, J. Fornwald, G. Scott, and L.A. Marshall, *The human polo-like kinase, PLK, regulates cdc2/cyclin B through phosphorylation and activation of the cdc25C phosphatase.* Cell Signal, 2000. **12**(6): p. 405-11.

37. Sumara, I., E. Vorlaufer, P.T. Stukenberg, O. Kelm, N. Redemann, E.A. Nigg, and J.M. Peters, *The dissociation of cohesin from chromosomes in prophase is regulated by polo-like kinase.* Molecular Cell, 2002. **9**(3): p. 515-525.

38. Hansen, D.V., A.V. Loktev, K.H. Ban, and P.K. Jackson, *Plk1 regulates activation of the anaphase promoting complex by phosphorylating and triggering SCFbetaTrCP-dependent destruction of the APC Inhibitor Emi1.* Mol Biol Cell, 2004. **15**(12): p. 5623-34.

39. Moshe, Y., J. Boulaire, M. Pagano, and A. Hershko, *Role of Polo-like kinase in the degradation of early mitotic inhibitor 1, a regulator of the anaphase promoting complex/cyclosome.* Proc Natl Acad Sci U S A, 2004. **101**(21): p. 7937-42.

40. Hanks, T.S. and K.A. Gauss, *Pleomorphic adenoma gene-like 2 regulates expression of the p53 family member, p73, and induces cell cycle block and apoptosis in human promonocytic U937 cells.* Apoptosis, 2012. **17**(3): p. 236-47.

41. Zheng, G., J. Ning, and Y.C. Yang, *PLAGL2 controls the stability of Pirh2, an E3 ubiquitin ligase for p53.* Biochem Biophys Res Commun, 2007. **364**(2): p. 344-50.
